# The transcription factor ORA59 represses hypoxia responses during *Botrytis cinerea* infection and reoxygenation

**DOI:** 10.1093/plphys/kiae677

**Published:** 2024-12-20

**Authors:** Luca Brunello, Alicja B Kunkowska, Emma Olmi, Paolo M Triozzi, Simone Castellana, Pierdomenico Perata, Elena Loreti

**Affiliations:** PlantLab, Institute of Plant Sciences, Sant’Anna School of Advanced Studies, Via Guidiccioni 10, 56010 San Giuliano Terme (Pisa), Italy; PlantLab, Institute of Plant Sciences, Sant’Anna School of Advanced Studies, Via Guidiccioni 10, 56010 San Giuliano Terme (Pisa), Italy; PlantLab, Institute of Plant Sciences, Sant’Anna School of Advanced Studies, Via Guidiccioni 10, 56010 San Giuliano Terme (Pisa), Italy; PlantLab, Institute of Plant Sciences, Sant’Anna School of Advanced Studies, Via Guidiccioni 10, 56010 San Giuliano Terme (Pisa), Italy; PlantLab, Institute of Plant Sciences, Sant’Anna School of Advanced Studies, Via Guidiccioni 10, 56010 San Giuliano Terme (Pisa), Italy; PlantLab, Institute of Plant Sciences, Sant’Anna School of Advanced Studies, Via Guidiccioni 10, 56010 San Giuliano Terme (Pisa), Italy; CNR, National Research Council, Institute of Agricultural Biology and Biotechnology, Via Moruzzi 1, 56124 Pisa, Italy

## Abstract

Transcription factors belonging to the large ethylene response factor (ERF) family are involved in plant responses to biotic and abiotic stresses. Among the ERFs, OCTADECANOID-RESPONSIVE ARABIDOPSIS 59 (ORA59) integrates ethylene and jasmonic acid signaling to regulate resistance to necrotrophic pathogens. The ERF group ERFVII encodes oxygen-labile proteins that are required for oxygen sensing and are stabilized by hypoxia established at the site of Botrytis (*Botrytis cinerea*) infection. Here, we show that ORA59 represses ERFVII protein activity to induce the expression of hypoxia-responsive genes in Arabidopsis (*Arabidopsis thaliana*). Moreover, inhibition of ethanol fermentation enhances plant tolerance to Botrytis, indicating a trade-off between the hypoxia and defense responses. In addition, ERFVII members and ORA59 are both involved in the downregulation of hypoxia-responsive genes during reoxygenation. Taken together, our results reveal that the ERFVII transcription factor–ORA59 module ensures that the multiple roles of ERFVII proteins are correctly balanced to favor plant tolerance to biotic or abiotic stresses.

## Teaser

A signaling module comprising the transcription factors ORA59 and ERFVII family members controls the hypoxia response during infection with the fungal pathogen *Botrytis cinerea* and during reoxygenation.

## Introduction

Botrytis (*Botrytis cinerea*) is a necrotrophic fungus responsible for a gray mold disease that kills its host plant cell and then grows on dead tissue. Botrytis can infect more than 200 dicot hosts, causing serious damage to crop productivity, which inevitably leads to economic losses (Williamson et al. 2007). The fact that Botrytis is considered one of the most dangerous pathogens worldwide has prompted many researchers to study the mechanism(s) utilized by plants to fight infection. Plants respond to pathogen attack through different biological responses at the physiological, biochemical, and molecular levels. Model organisms such as Arabidopsis (*Arabidopsis thaliana),* have been used to reveal many aspects of the plant-Botrytis interaction.

The plant's response to Botrytis involves phytohormones, such as salicylic acid (SA), jasmonic acid (JA), ethylene (ET), and abscisic acid (ABA). In Arabidopsis, the response against biotrophic and hemibiotrophic pathogens is generally mediated by SA (Wildermuth et al. 2001), whereas JA and ET play a role against necrotrophic pathogens (Lorenzo et al. 2003; Thaler et al. 2004). The resistance of Arabidopsis plants defective in ETHYLENE INSENSITIVE 2 to Botrytis is impaired, suggesting that functional ethylene signal transduction is required to fight pathogen infection (Thomma et al. 1999). Several ethylene response factors (ERFs) are involved in plant stress responses, including Botrytis attack (Thaler et al. 2004; Broekgaarden et al. 2015). Among them, OCTADECANOID-RESPONSIVE ARABIDOPSIS 59 (ORA59) has been identified as a regulator of Botrytis resistance, given that transgenic plants overexpressing *ORA59* exhibit increased resistance to Botrytis, whereas *ORA59* gene-silenced plants are more susceptible (Pré et al. 2008). Furthermore, *ORA59* gene expression is synergistically induced by JA and ET, suggesting that ORA59 plays a role in their signaling pathways (Pré et al. 2008).

The involvement of ERFs during pathogen infection has also been demonstrated by the interaction between ORA59 and RELATED TO AP2.3 RAP2.3 (Kim et al. 2018). Both the ORA59 and RAP2.3 proteins were shown to be required for Arabidopsis resistance to the necrotrophic pathogen *Pectobacterium carotovorum* (Kim et al. 2018). Notably, ORA59 interacts with RAP2.3 in the nucleus, although the interaction of ORA59 and RAP2.3 is not required for nuclear translocation (Kim et al. 2018). Although both ORA59 and RAP2.3 are required for Arabidopsis defense responses, it is unclear whether all ERFVIIs potentially interact with ORA59 and what consequences these interactions might have on the primary role of ERFVIIs, namely, the induction of hypoxia-responsive genes (*HRGs*) (Gasch et al. 2016). Together with RAP2.12, RAP2.2, and hypoxia-responsive ERF (HRE)1 and 2, RAP2.3 belongs to subgroup VII of ERFs. In addition to their well-studied role in hypoxia signaling (Loreti et al. 2016; Loreti and Perata 2023), ERFVII proteins play an emerging role in the plant response to pathogens (Zhao et al. 2012; Gravot et al. 2016; Kim et al. 2018; Vicente et al. 2019; Valeri et al. 2021). There is evidence that *35S:RAP2.2* Arabidopsis plants are more tolerant to Botrytis infection, whereas the mutant line *rap2.2–3* is more susceptible, suggesting that ERFVIIs play a role in Botrytis infection (Zhao et al. 2012). Overexpression of ERF72 (RAP2.3) increases resistance to Botrytis in Arabidopsis, suggesting that both RAP2.3 and RAP2.2 may play a role in Botrytis resistance (Li et al. 2022).

ERFVII transcription factors play a pivotal role in the sensing of low oxygen and nitric oxide (NO) (Gibbs et al. 2011, 2014; Licausi et al. 2011). RAP-type ERFVIIs are constitutively expressed under normal oxygen availability, but their *N*-terminal cysteine residues (after Met removal) are exposed and oxidized by plant cysteine oxidase (PCO) (Weits et al. 2014; White et al. 2018), which channels these ERF-VII proteins to the proteasome (Varshavsky 2011) for degradation (the *N*-degron pathway). When low oxygen occurs, PCOs are not able to oxidize the *N*-terminal cysteine residues, and ERFVIIs are stabilized and migrate into the nucleus, triggering the transcription of *HRGs* (Gasch et al. 2016).

Botrytis infection causes a drop in oxygen level in the Arabidopsis leaf regions undergoing fungal growth, due to increased respiration rate of the fungus (Valeri et al. 2021). Interestingly, using a system in which Botrytis can elicit responses in Arabidopsis cells without causing an infection, oxygen consumption was strongly increased, indicating that the metabolism of the host was stimulated upon elicitation by Botrytis (Veillet et al. 2017). Also the pathogen elicitor flagellin22 induces a drop in oxygen concentration in Arabidopsis leaves (Valeri et al. 2021).

The link between Botrytis infection and hypoxia has been described in Arabidopsis leaves (Valeri et al. 2021). In fact, the role of ERFVII proteins during Botrytis infection predicts that these oxygen-labile proteins are stabilized during infection. The establishment of local hypoxia leads to the stabilization and relocalization of RAP2.12, (Gibbs et al. 2011; Licausi et al. 2011). However, the consequences of ORA59 interaction with some ERFVII proteins are largely unknown.

Here, we show that ERFVIIs are involved in plant defense pathways, with ORA59 representing a counterpart in the ERFVII-dependent regulation of the hypoxic response. The ORA59-ERFVII module is also involved in the dampening of the hypoxic response during plant reoxygenation, indicating that the role of ORA59 goes beyond the responses to necrotrophic fungi and represents a player in the regulation of gene expression during hypoxia.

## Results

### Only a subset of hypoxia-responsive genes is activated during Botrytis infection

**Figure 1.**
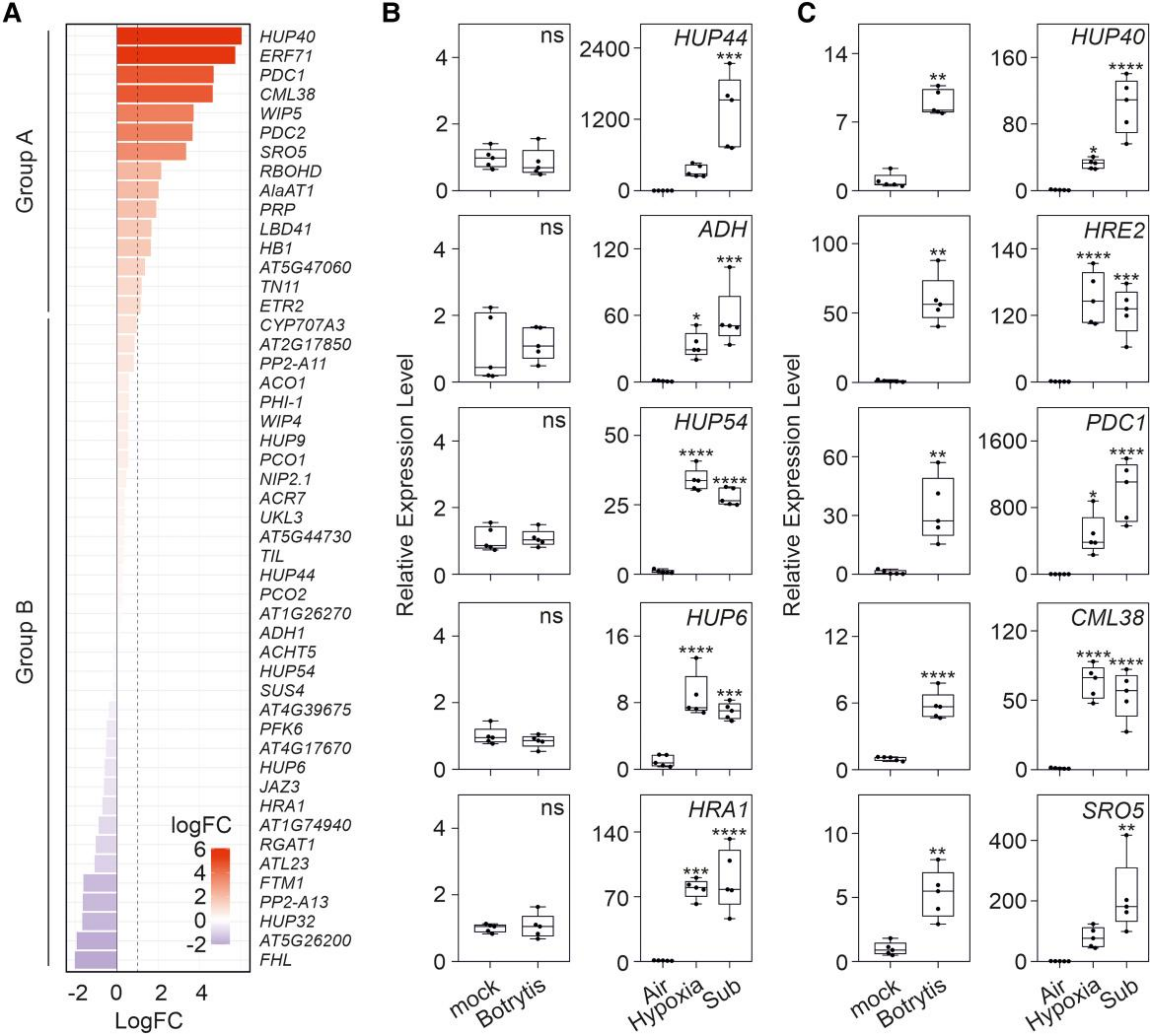
Expression of hypoxia-responsive genes during Botrytis infection **A)** bar plot of log_2_ fold change (log_2_FC) expression values for core anaerobic genes after 48 h from the inoculation with Botrytis. Genes are sorted in descending order, with a dashed line indicating the threshold of log_2_FC = 1, representing significantly upregulated genes in adult Arabidopsis plants rosette 48 h post infection. **B)** Expression analysis of Group B core anaerobic genes after 48 h from infection with Botrytis and upon low-oxygen stresses. The treatments were 4 h of gaseous hypoxia and 4 h of submergence. Data (*n* = 5) are relative to the mock group for Botrytis and to the air group for hypoxia/submergence treatments, both set to 1. **C)** Expression analysis of Group A core anaerobic genes after 48 h from infection with Botrytis and upon low-oxygen stress. The treatments were as in panel **B)**. Statistically significant differences in **B)** and **C)** are indicated by the asterisks (Student *t*-test, unpaired comparison was used for mock to Botrytis comparison, one-way ANOVA followed by Dunnett's multiple comparisons test was used for air to hypoxia and submergence comparisons. * = *P* < 0.05; ** = *P* < 0.01; *** = *P* < 0.001; **** = *P* < 0.0001). Lines within the boxes indicate the median, while the bottom and top of each box denote the first and third quartile, respectively, the dots represent the single data points and whiskers denote the min/max values.

To explore the extent of *HRGs* induction in response to hypoxia caused by Botrytis infection, we focused on the 49 core anaerobic genes (Mustroph et al. 2010). To our surprise, only few of these genes were induced after Botrytis infection (Fig. 1A, Group A), while a large group of these *HRGs* was not induced or even slightly repressed after infection (Fig. 1A, Group B). This is counterintuitive, given that Botrytis causes hypoxia (Valeri et al. 2021). The *HRGs* are all induced by hypoxia (Mustroph et al. 2010) and are transcriptionally controlled by ERFVIIs (Gasch et al. 2016). We checked the expression of *HRGs* belonging to the 2 groups in plants subjected to Botrytis, hypoxia, and submergence. The expression of alcohol dehydrogenase (*ADH*), a classical marker of hypoxia responses at the transcriptional level, was not induced by Botrytis (Fig. 1B), in agreement with Valeri et al. (2021). Under hypoxia and submergence, *ADH* was instead induced, as expected (Fig. 1B). A similar pattern of expression was observed for other Group B genes, such as *HYPOXIA RESPONSE UNKNOWN PROTEIN 44* (*HUP44*), *HUP54*, *HUP6*, and *HYPOXIA RESPONSE ATTENUATOR1* (*HRA1*). The expression of other *HRGs* belonging to Group A, namely, *HUP40*, *HYPOXIA RESPONSIVE ERF2* (*HRE2*), *PIRUVATE DECARBOXYLASE1* (*PDC1*)*, CALMODULIN-LIKE 38* (*CML38*), and *SIMILAR TO RCD ONE 5* (*SRO5)* was instead strongly induced by Botrytis (Fig. 1C).

These findings suggest the suppression of ERFVII activity for a large group of *HRGs*, despite the evidence of the stabilization of these transcription factors during Botrytis-induced hypoxia (Valeri et al. 2021).

### ORA59 represses the ERFVII-dependent induction of *HRGs*

A large set of *HRGs* is not induced by Botrytis (Fig. 1A; Group B), despite hypoxia and concomitant ERFVII stabilization occurring in Botrytis-infected leaves (Valeri et al. 2021). The expression of *HRA1*, which is a known repressor of ERFVII activity (Giuntoli et al. 2014), was not induced by Botrytis (Fig. 1B), thus potentially rule out its involvement in repressing ERFVIIs-dependent *HRGs* induction.

**Figure 2.**
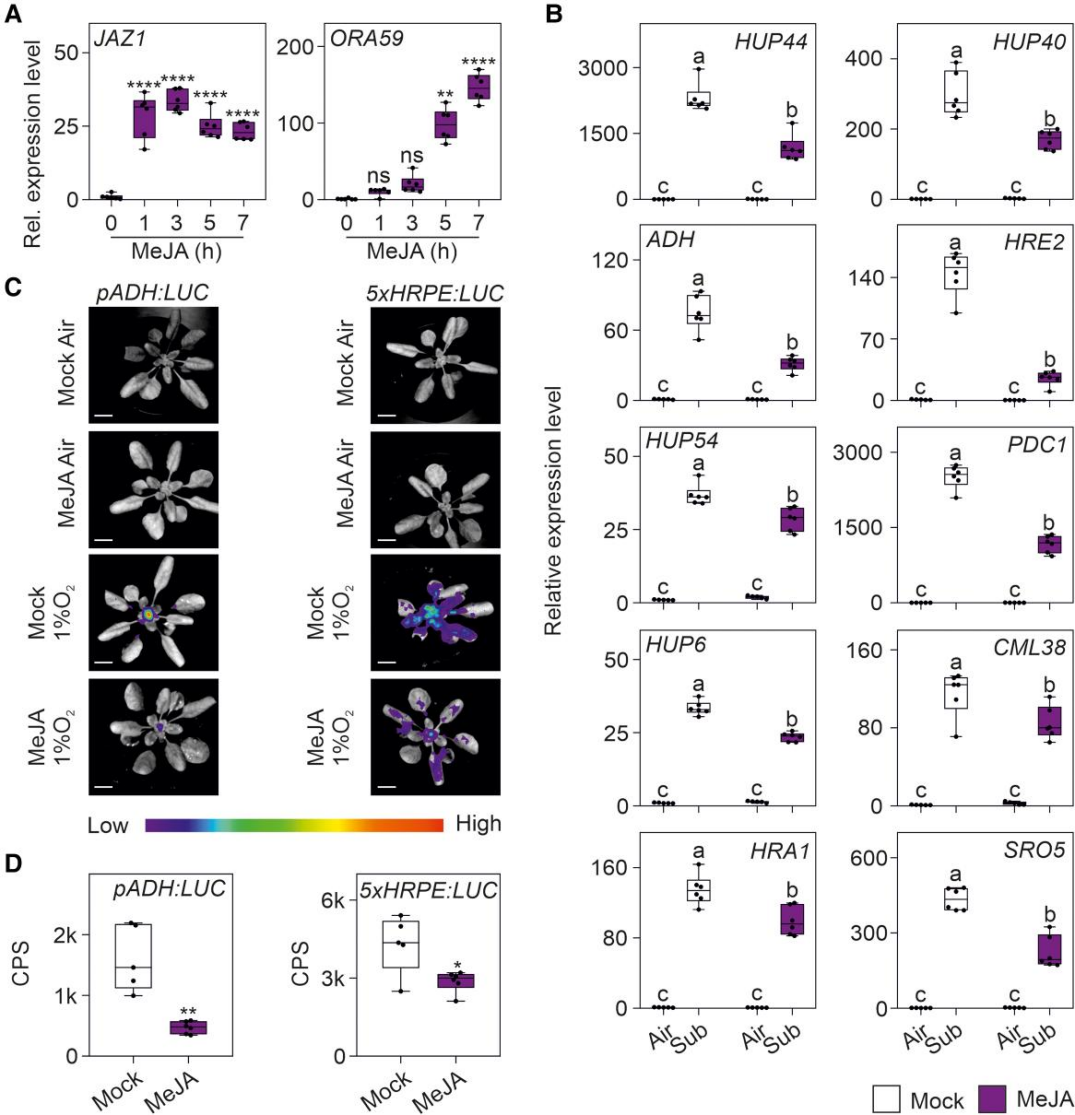
Effects of jasmonate treatments on the expression of *HRGs*. **A)** Time-course expression analysis of jasmonate-responsive marker genes. Statistically significant differences are indicated by asterisks after Kruskal–Wallis test followed by Dunn's multiple comparisons test (* = *P* < 0.05; ** = *P* < 0.01; *** = *P* < 0.001; **** = *P* < 0.0001). Data (*n* = 6) are relative to time 0 group set to 1. **B)** Expression analysis of Group B and A core anaerobic genes in adult Arabidopsis plants rosette pretreated with 50 µM MeJA for 7 h and then submerged in the dark for 2 h. Different letters indicate significant difference (*P* < 0.05) after two-ways ANOVA followed by Tukey's post-hoc test. Data (*n* = 6) are relative to the Col-0 control (air) not pretreated with 50 μM MeJA, set to 1. **C)** Representative images of LUC activity in *pADH:LUC* and *5xHRPE:LUC* plants after 7 h incubation with 50 µM MeJA and 2 h of gaseous hypoxia. The scale bar is 1 cm. **D)** Quantification of LUC activity (counts per second, CPS) of *pADH:LUC* and *5xHRPE:LUC* reporter lines showed in **C)** (*n* ≥ 5). Quantification of CPS was carried out using the IndiGO software, by selecting a fixed area around the plants. Statistically significant differences are indicated by the asterisks (Student *t*-test, unpaired comparison; * = *P* < 0.05; ** = *P* < 0.01; *** = *P* < 0.001; **** = *P* < 0.0001). Lines within the boxes indicate the median, while the bottom and top of each box denote the first and third quartile, respectively, the dots represent the single data points and whiskers denote the min/max values.

Convergence of the hypoxic pathway with the plant's response to Botrytis is represented by increased levels of JA. JA-dependent responses are important for activating the plant defense pathway in response to Botrytis, but evidence for an increased JA level is also available for the response of Arabidopsis to hypoxia (Yuan et al. 2017). Arabidopsis plants treated with methyl-jasmonate (MeJA) clearly expressed the JA-responsive genes *JAZ1* and *ORA59* (Fig. 2A) and showed a lower induction of *HRGs* in submerged plants (Fig. 2B). This indicates that JA reduces the induction of *HRGs*. Imaging of luciferase (LUC) activity in plants that were treated under hypoxia after an exogenous MeJA treatment highlighted a clear reduction of luminescence signal from hypoxia-responsive *pADH:LUC* as well as in the 5-time repeated hypoxia-responsive promoter elements (5xHRPE) *LUC* reporter lines (*5xHRPE:LUC*; Fig. 2C and D). ERFVIIs directly interact with the HRPE (Gasch et al. 2016), indicating that the MeJA repression is due to reduced ability of ERFVIIs to bind to the hypoxia-responsive promoter elements.

**Figure 3.**
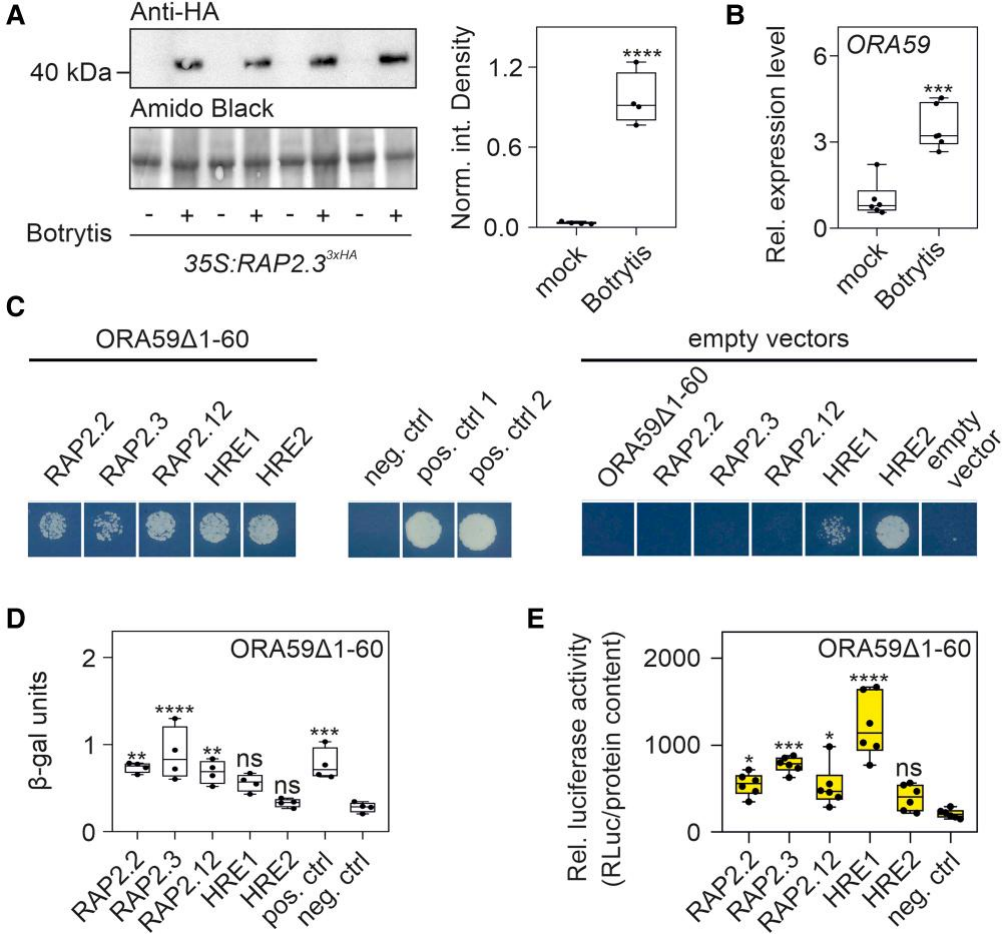
ORA59 interacts with the ERFVIIs. **A)** Western blot analysis of RAP2.3^3xHA^ abundance in adult Arabidopsis plants leaves 48 h post infection with Botrytis followed by the quantification of the signal carried out using ImageLab^TM^ software (BioRad). 40 μg of protein was loaded for each sample. Four biological replicates are shown. **B)** Expression analysis of *ORA59* in Arabidopsis rosettes 48 h post infection. Data (*n* = 6) are relative to the mock group set to 1. Statistically significant differences are indicated by the asterisks (Student *t*-test, unpaired comparison; * = *P* < 0.05; ** = *P* < 0.01; *** = *P* < 0.001; **** = *P* < 0.0001). **C)** Yeast two-hybrid assay between the 5 ERFVIIs and ORA59 (N-terminal 60 amino acids truncated, ORA59Δ1–60). **D)** As in **C)**, followed by a β-galactosidase assay (CPRG) test (*n* = 4). **E)** Split-LUC assay of ORA59 fused to the N-terminal part of the firefly LUC and the ERFVIIs members (and negative control, HOL2) fused to the C-terminal part of the firefly LUC each (*n* = 6). For **D)** and **E)**, statistically significant differences are indicated by asterisks after a one-way ANOVA followed by Dunnett's multiple comparisons test (* = *P* < 0.05; ** = *P* < 0.01; *** = *P* < 0.001; **** = *P* < 0.0001). Lines within the boxes indicate the median, while the bottom and top of each box denote the first and third quartile, respectively, the dots represent the single data points and whiskers denote the min/max values.

Treatment with Botrytis leads to the stabilization and nuclear localization of ERFVII (Valeri et al. 2021). Evaluation of RAP2.3^3xHA^ protein levels by immunoblot confirmed ERFVII stabilization after Botrytis infection (Fig. 3A). Botrytis also induces *ORA59* (Fig. 3B), which interacts with RAP2.3 (Kim et al. 2018). Given that the ERFVII family is composed of 5 members, we tested whether, in addition to RAP2.3, ORA59 interacts with other members of this family. A Y2H assay showed that ORA59 interacts with all ERFVIIs except for HRE1 and HRE2 (Fig. 3C), a result confirmed by the chlorophenol red-β-D-galactopyranoside (CPRG) assay (Fig. 3D). A split-LUC assay also indicated that HRE2 does not interact with ORA59, while HRE1 had a positive interaction in this assay (Fig. 3E).

**Figure 4.**
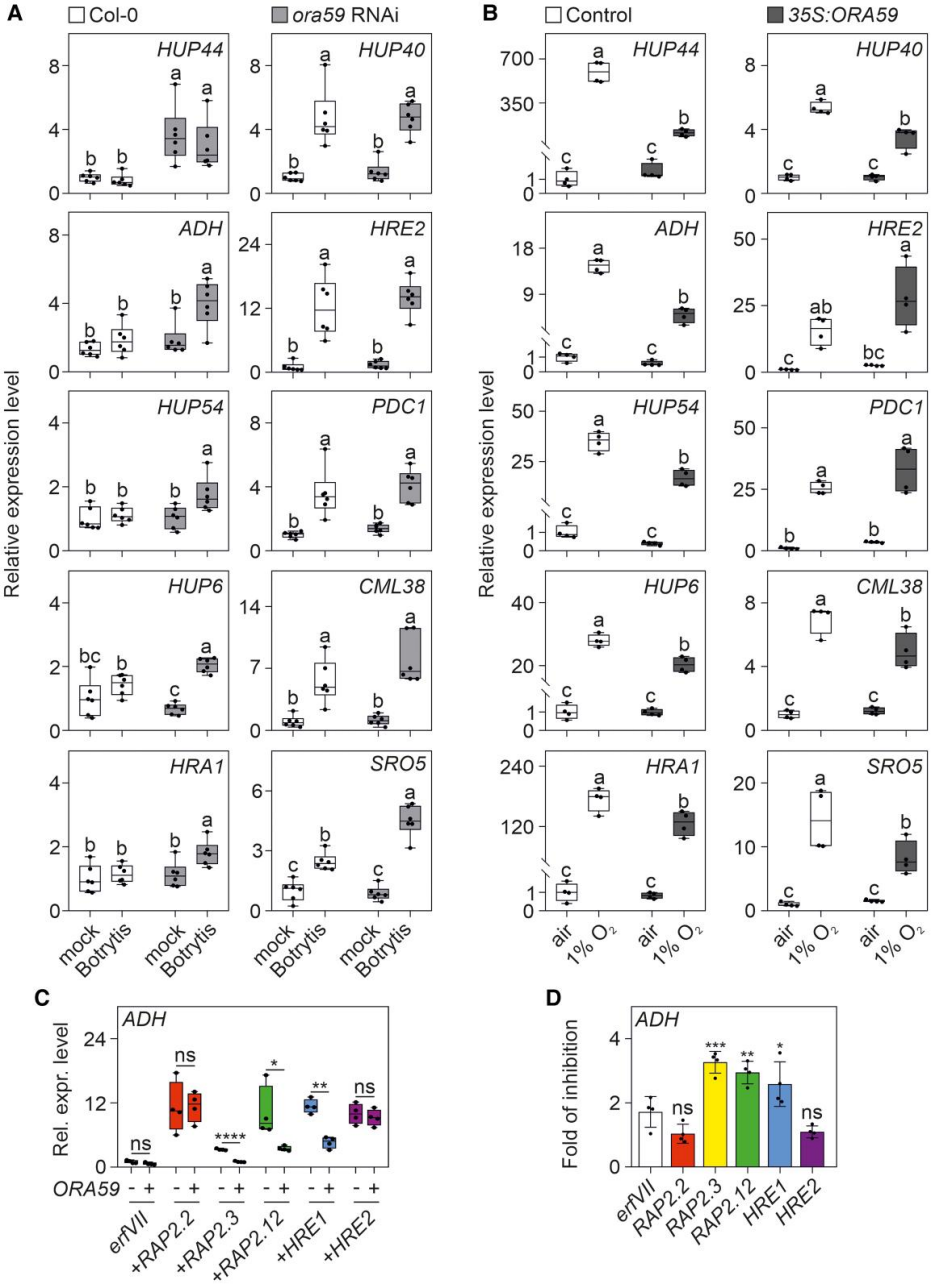
ORA59 is a repressor of HRGs expression. **A)** Expression analysis of Group B and A core anaerobic genes in Col-0 and *ora59* RNAi adult Arabidopsis rosette 48 h after inoculation with Botrytis. Data (*n* = 6) are relative to the Col-0 mock set to 1. **B)** Expression of Group B and A core anaerobic genes in Col-0 protoplasts transformed with *35S:ORA59* and treated with gaseous hypoxia for 2 h. Data (*n* = 4) are relative to the Col-0 untransformed control (air) set to 1. For **A)** and **B)**, different letters indicate significant difference (*P* < 0.05) after two-ways ANOVA followed by Tukey's post-hoc test. **C)** Expression of *ADH* in *erfVII* protoplasts treated with gaseous hypoxia for 2 h. Protoplasts were transformed with each of the ERFVIIs singularly, under the control of the cauliflower mosaic virus 35S promoter, and with or without ORA59 driven by the same promoter. Statistically significant differences are indicated by asterisks after multiple unpaired *t*-test (* = *P* < 0.05; ** = *P* < 0.01; *** = *P* < 0.001; **** = *P* < 0.0001). Data (*n* = 4) are relative to the *erfVII* untransformed group, set to 1. **D)** Inhibition of ERFVII-dependent expression of *ADH* by *ORA59* in *erfVII* protoplasts. Data are the same as in Fig. 4C and are mean (*n* = 4) ±SD. Statistically significant differences are indicated by asterisks after a one-way ANOVA followed by Dunnett's multiple comparisons test (* = *P* < 0.05; ** = *P* < 0.01; *** = *P* < 0.001; **** = *P* < 0.0001). In box-plots, lines within the boxes indicate the median, while the bottom and top of each box denote the first and third quartile, respectively, the dots represent the single data points and whiskers denote the min/max values.

We then used an *ORA59* RNAi line (*ora59*), which is impaired in ORA59 transcriptional activity, as shown by its inability to activate its target gene *PLANT DEFENSIN 1.2* (*PDF1.2*) (Supplementary Fig. S1). Notably, *HRGs* belonging to Group B (Fig. 1A) were induced when the *ora59* line was challenged with the pathogen (Fig. 4A, left column), indicating that ORA59 may act as a repressor of ERFVII activity. *HUP44* was expressed at a higher level in *ora59* even when not challenged by Botrytis (Fig. 4A). *HRGs* belonging to Group A (Fig. 1A) were, as expected, induced by Botrytis also in the wild-type, with no differences detected in *ora59* (Fig. 4A, right column). Next, we overexpressed *ORA59* in Arabidopsis protoplasts (Fig. 4B) and examined the induction of *HRGs* belonging to Group B (Fig. 1A) by hypoxia. The expression of all tested *HRGs* was dampened by the overexpression of *ORA59* (Fig. 4B, left column), thus providing direct evidence of a negative role of ORA59 in the induction of *HRG*s. Also, genes belonging to Group A (Fig. 1A) were negatively affected, except for *PDC1* and *HRE2* (Fig. 4B). In this context, it is notable that the only ERFVII protein that does not interact with ORA59, namely, HRE2, would be theoretically sufficient to upregulate *HRGs*, but HRE2 is a poor transcriptional activator of *HRGs* (Gasch et al. 2016). To better understand the impact of ORA59 on the activity of each individual ERFVII, we used protoplasts derived from the *erfVII* mutant complemented with each individual ERFVII, which was stabilized by hypoxia treatment and challenged with ORA59 (Fig. 4C). All 5 ERFVIIs restored *ADH* induction in the *erfVII* mutant (Fig. 4C). However, the activation of *ADH* by RAP2.3, RAP2.12, and HRE1 was repressed in presence of ORA59 overexpression, while the activity of RAP2.2 and HRE2 was unaffected (Fig. 4C and D).

### Pyruvate decarboxylase and ADH activities are detrimental to tolerance to Botrytis

**Figure 5.**
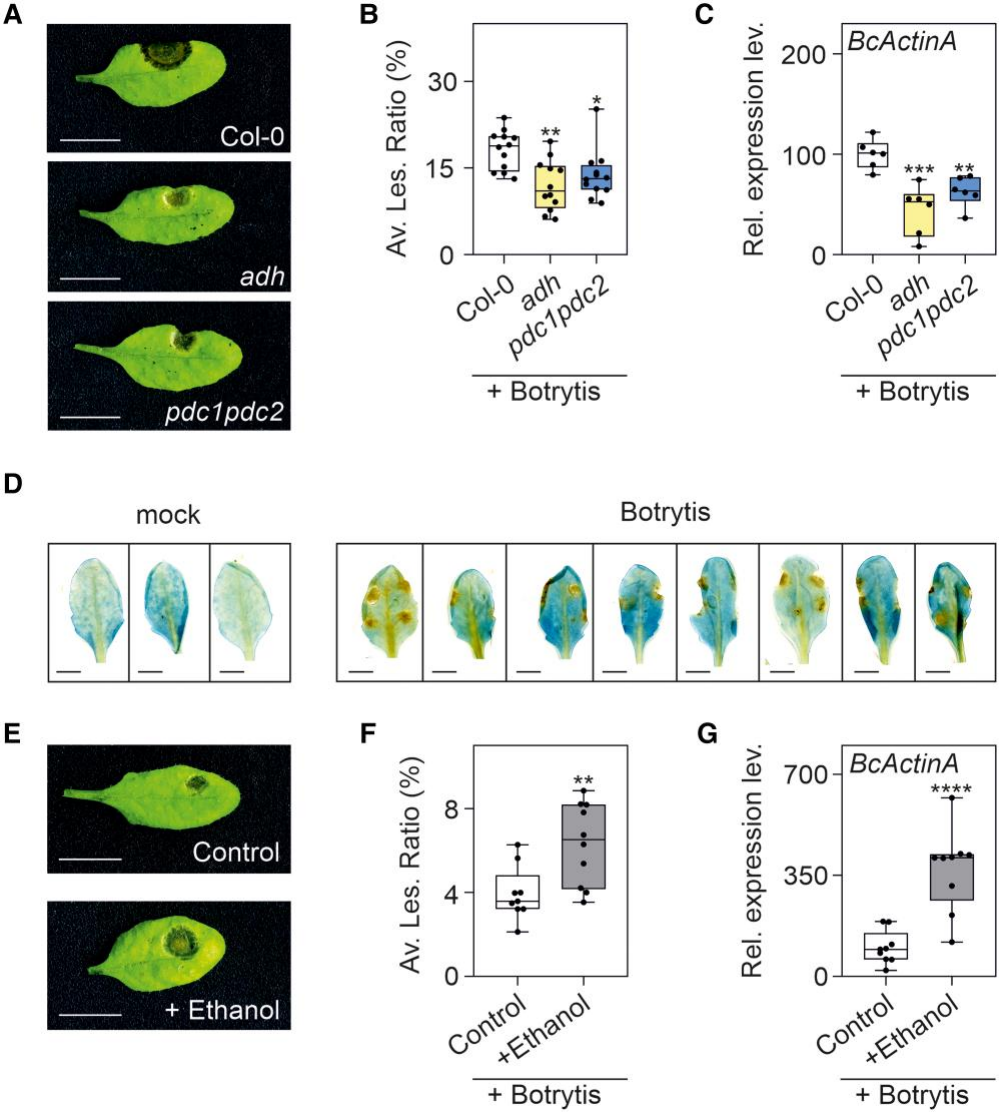
Production of ethanol is detrimental or Arabidopsis tolerance to Botrytis. **A)** Tolerance after Botrytis infection in Col-0, *adh*, *pdc1pdc2* double mutants. The images show the lesion area 48 h after the inoculation. The scale bar is 1 cm. **B)** Percentage of lesion area normalized on the leaf size presented in a boxplot. Each dot represents the average value of a plant (*n* = 12) (5 different infected leaves on each plant). **C)** Expression analysis in adult Arabidopsis rosette of *B. cinerea* actin A (*BcActinA*) as marker for the presence of the fungi. Data (*n* = 6) are relative to Col-0 infected group set to 100 for *BcActinA*. For **B)** and **C)**, statistically significant differences are indicated by asterisks after a one-way ANOVA followed by Dunnett's multiple comparisons test (* = *P* < 0.05; ** = *P* < 0.01; *** = *P* < 0.001; **** = *P* < 0.0001). **D)** Transgenic line expressing GUS under the control of the *ADH* promoter (*pADH:GUS*). GUS staining was performed 48 h after the inoculation with Botrytis. The scale bar is 0.5 cm. **E)** Tolerance after Botrytis infection in Col-0 plants. Spores used in the infection were resuspended in either PDB (Control) or PDB added with 100 mM Ethanol. The images show the lesion area 48 h after the inoculation. The scale bar is 1 cm. **F)** Percentage of lesion area normalized on the leaf size presented in a boxplot. Each dot represents the average value of a plant (*n* ≥ 9) (5 different infected leaves on each plant). **G)** Expression analysis in adult Arabidopsis rosette of *B. cinerea* actin A (*BcActinA*) as marker of the fungi presence. Data (*n* = 9) are relative to Col-0 infected group set to 100 for *BcActinA*. For **F)** and **G)**, statistically significant differences are indicated by the asterisks (Student *t*-test, unpaired comparison; * = *P* < 0.05; ** = *P* < 0.01; *** = *P* < 0.001; **** = *P* < 0.0001). Lines within the boxes indicate the median, while the bottom and top of each box denote the first and third quartile, respectively, the dots represent the single data points and whiskers denote the min/max values.

ERFVIIs are required for tolerance to Botrytis infection (Valeri et al. 2021), but the induction of *ORA59* during infection likely dampens ERFVII action on *HRGs* (Fig. 4), suggesting that activation of the plant's anaerobic response is detrimental for tolerance to the pathogen. We tested this hypothesis by using 2 mutants defective in the metabolic steps required for energy production under hypoxia, namely, the alcoholic fermentation enzymes pyruvate decarboxylase (PDC) and ADH (Mithran et al. 2014). Both the *pdc1pdc2* and *adh* were more tolerant to Botrytis, as shown by the smaller lesion size (Fig. 5A and B) and the lower fungal presence in the leaves, as indicated by the *B. cinerea* actin A (*BcActinA*) expression level (Fig. 5C). Given that Botrytis fails to induce *ADH* expression, it is puzzling that the lack of ADH in the *adh* mutant impacts on Botrytis tolerance. However, both PDC and ADH are present in Arabidopsis plants even under aerobic conditions (Mithran et al. 2014). We checked the presence of ADH by using a *pADH:GUS* line and confirmed that ADH is present in aerobic leaves independently of Botrytis presence (Fig. 5D). We then verified if the product of the pathway sequentially requiring PDC and ADH, namely ethanol, influences the infection by Botrytis. The infection was performed in the presence of exogenously applied ethanol, resulting in a much lower tolerance to Botrytis (Fig. 5E and F), thus confirming that production of ethanol by the combined action of PDC and ADH is detrimental for the plant tolerance to Botrytis. Leaves treated with ethanol-only did not show any toxicity symptoms, in line with the absence of ethanol toxicity per se (Supplementary Fig. S2). The stronger expression of *BcActinA* confirms the higher presence of Botrytis when ethanol was present (Fig. 5G).

### The interplay between the ERFVIIs and ORA59 influences the reoxygenation phase after hypoxia

**Figure 6.**
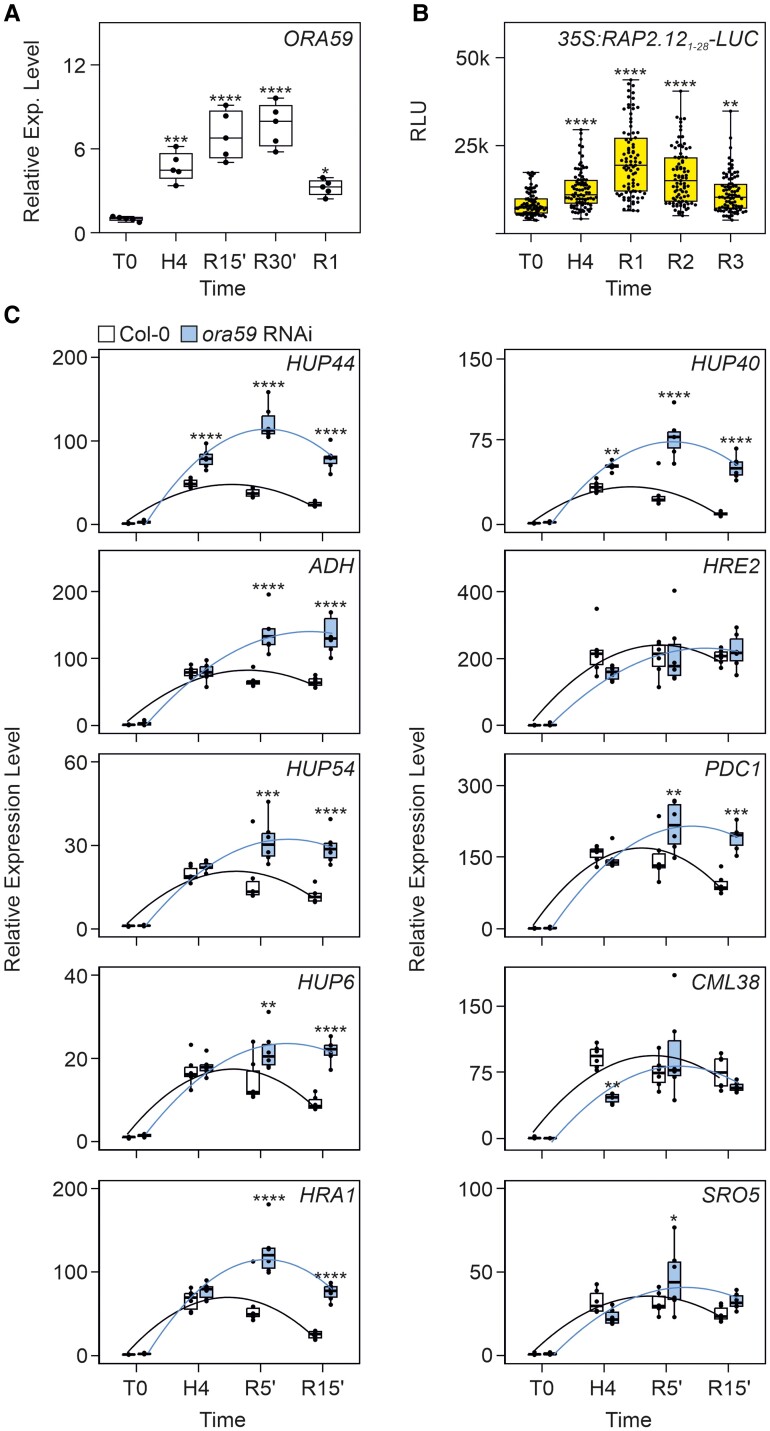
The interplay between the ERFVIIs and ORA59 influences the reoxygenation phase after hypoxia. **A)** Expression analysis of *ORA59* in Arabidopsis 5-d old seedlings during recovery in air for 5′ (R5′), 15′ (R15′), 30′ (R30′), and 1 h (R1) after 4 h of gaseous hypoxia (H4). Data (*n* = 5) are relative to aerobic (T0) seedlings set to 1. **B)** LUC activity in *35S:RAP2.12_1–28_-LUC* 7-d old seedling during post-hypoxia recovery in air for 1 h (R1), 2 h (R2), and 3 h (R3). For **A)** and **B)**, statistically significant differences (*n* = 96) are indicated by the asterisks after a one-way ANOVA followed by Dunnett's multiple comparisons test (* = *P* < 0.05; ** = *P* < 0.01; *** = *P* < 0.001; **** = *P* < 0.0001). **C)** Expression analysis of Group B and A core anaerobic genes during recovery after 4 h of gaseous hypoxia in Col-0 and *ora59* RNAi 5-d old seedlings. Curved lines represent polynomial fits passing through the mean (*n* = 6) of each group of observations, providing a visual representation of the trend within each genotype. Statistically significant differences are assessed using two-ways ANOVA followed by Sidak's multiple comparisons test (* = *P* < 0.05; ** = *P* < 0.01; *** = *P* < 0.001; **** = *P* < 0.0001). Lines within the boxes indicate the median, while the bottom and top of each box denote the first and third quartile, respectively, the dots represent the single data points and whiskers denote the min/max values.

Reoxygenation after hypoxia in Arabidopsis results in the rapid accumulation of JA and increased transcript levels of JA biosynthesis genes (Yuan et al. 2017), which suggests that *ORA59* is induced as well. Indeed, we verified that *ORA59* is induced during hypoxia and even stronger during the reoxygenation phase (Fig. 6A). *HRGs* expression during the recovery is believed to follow the destabilization of ERFVII triggered by oxygen, which is however quite slow, as measured from the number of nuclei with ERFVII localization during and after reoxygenation (Kosmacz et al. 2015). We confirmed that ERFVII stability is retained for several hours during reoxygenation, with a high RAP2.12_2–28_:LUC level even 3 h after reoxygenation (Fig. 6B). However, already after 15 min of reoxygenation, *HRGs* expression declines and progressively reaches normoxic levels after 2 h of reoxygenation (Kosmacz et al. 2015). We tested if ORA59 could be involved in the modulation of *HRGs* during the recovery phase. The results showed that in the absence of ORA59 (in the *ora59* RNAi line) the recovery (downregulation of HRGs) is much slower than in the wild-type (Fig. 6C). Not only the recovery is slower, but the expression of *HRGs* in *ora59* significantly increases even during the reoxygenation phase, indicating that ORA59 is required to ensure that *HRGs* are not expressed when oxygen presence is resumed after hypoxia.

## Discussion

The ERFVII proteins RAP2.2 and RAP2.3 have been identified as taking part in plant responses to necrotrophic fungi (Zhao et al. 2012; Kim et al. 2018). The expression of *RAP2.2* is induced by ethylene (Hinz et al. 2010), and RAP2.3 responds positively to ethylene, JA, and ABA (Kim et al. 2018); thus, these genes are potentially involved in the canonical pathways of plant defense. However, because these proteins are oxygen labile (Gibbs et al. 2011; Licausi et al. 2011), hypoxia must be present at the site of necrotroph infection for them to play a role in plant defense.

Hypoxia establishment during Botrytis infection has been confirmed by experimental evidence of ERFVIIs nuclear localization (Valeri et al. 2021) and stabilization (Fig. 3A). The fact that RAP2.3 (group VII ERF) physically interacts with ORA59 (group IX ERF) (Kim et al. 2018) suggests that ERFVII transcription factors may converge with the JA pathway. Given the crucial role of ERFVII proteins in hypoxia responses and tolerance, we explored their function in the context of hypoxia generated during pathogen infection. To our surprise, most of the core anaerobic genes (Mustroph et al. 2010) were not induced by Botrytis infection (Fig. 1), despite the strong hypoxic zone generated at the lesion site (Valeri et al. 2021). Notably, we found that the lack of induction of *HRGs* after Botrytis infection was due to the concomitant presence of ORA59, which interacts with most ERFVIIs (Fig. 3C to E) and represses the induction of *HRGs* (Fig. 4). Specifically, ORA59 repressed the effects of RAP2.3, RAP2.12, and HRE1 on *ADH* expression but not those of RAP2.2 or HRE2 (Fig. 4D). This explains why the repression exerted by ORA59 in the wild-type background (Fig. 4B) was not as strong as that observed in the context of *erfVII* protoplasts complemented with each individual ERFVII (Fig. 4C and D). The lack of repression by ORA59 on the HRE2-driven induction of *ADH* is likely explained by the lack of ORA59-HRE2 interaction (Fig. 3C to E), while in the case of RAP2.2, the explanation is still unknown and may reside in a conformational difference in the interaction with ORA59. The negative impact of ORA59 on *HRGs* expression was not limited to the response to Botrytis but also to hypoxia, as the expression of *HRGs* was dampened under hypoxia when *ORA59* was overexpressed (Fig. 4B). Similarly, a plant's MeJA pretreatment followed by submersion led to a reduced expression of *HRGs* (Fig. 2B).

When Botrytis infects Arabidopsis leaves, greater oxygen consumption is observed in infected leaves, which is due to fungal respiration (Valeri et al. 2021), although infection per se is not required (Veillet et al. 2017) and also the pathogen elicitor flagellin22 induces a drop in oxygen concentration in Arabidopsis leaves (Valeri et al. 2021). This results in the generation of a hypoxic niche coinciding with the leaf lesion generated by Botrytis. Hypoxia-driven stabilization of RAP2.12 activates the SA pathway through *SALICYLIC ACID INDUCTION DEFICIENT 2* (*SID2*) transcriptional activation (Koo et al. 2024). ERFVIIs are therefore involved in the antagonistic interplay between the JA pathway (with ORA59 as the effector) and SA biosynthesis as previously described (Koo et al. 2024).

Given that the activation of fermentative metabolism via ERFVII factors is crucial for plant survival under hypoxia, it would be tempting to speculate that the inhibition of *ADH* induction by ORA59 in hypoxic lesions caused by Botrytis would lead to a hypersensitive response-like effect that would be favorable for Botrytis (Govrin and Levine 2000). Interestingly, the opposite is true, with 2 independent fermentative mutants (*adh* and *pdc1pdc2*) severely impaired in ethanol synthesis (Bui et al. 2019), showing enhanced tolerance to Botrytis (Fig. 5A to C). This suggests that ethanol production in the hypoxic lesion area is detrimental to tolerance, possibly because of the effects of ethanol on the expression of a large number of defense-related genes, including a large number of SA-related genes (Yuan et al. 2020). Remarkably, exogenous ethanol application at the site of infection leads to lower tolerance to Botrytis (Fig. 5E to G). In this context, the repression of ERFVII activity on *HRGs* (and therefore on fermentative metabolism) exerted by ORA59 contributes to plant tolerance to Botrytis. Both ADH and PDC activities are required for optimal plant development, even under aerobic conditions (Ventura et al. 2020). Repression of ADH and PDC is therefore restricted to conditions inducing ORA59 expression, namely during pathogen infection and recovery from a previous hypoxic condition.

Although Botrytis-induced hypoxia and subsequent ERFVII stabilization are presumably detrimental to plant tolerance to Botrytis because of the positive effect of ERFVII on the SA pathway (Koo et al. 2024), the *erfVII* mutant is less tolerant to Botrytis (Valeri et al. 2021), as were the *rap2.2* and *rap2.3* mutant plants (Zhao et al. 2012; Li et al. 2022). ERFVII action thus appears required for Botrytis resistance, beyond the activation of *HRGs*, an hypothesis requiring further studies.

Our results indicated that the ERFVII-ORA59 module plays a pivotal role in orchestrating a balanced plant response toward enhanced Botrytis tolerance, via repression of the fermentative metabolism, which is detrimental for Botrytis tolerance. The interplay between the ERFVII and ORA59 expands to the modulation of HRGs expression during the post-hypoxic phase. Reoxygenation is characterized by a burst in JA synthesis (Yuan et al. 2017), which triggers ORA59 expression (Fig. 6A), positively contributing to the reduction in *HRGs* expression in the context of high ERFVII stability (Fig. 6B) and their presence in the nuclei of plants, which are no more under hypoxia (Kosmacz et al. 2015). The ORA59-ERFVII module represents a player in the regulation of hypoxic signaling.

## Materials and methods

### Plant material and growth conditions

The *A. thaliana* Columbia-0 (Col-0) ecotype was used as the wild-type in all the experiments. The following transgenic lines were used: *erfVII* (Marín-de La Rosa et al. 2014), *pADH:GUS* (Ventura et al. 2020), 35S:*RAP2.3^3xHA^* (Gibbs et al. 2014), and a reporter line bearing the first 28 amino acids of RAP2.12 fused to the firefly LUC coding sequence (*35S:RAP2.12_1–28_-LUC*) (Weits et al. 2014), the *ora59* RNAi line (Pré et al. 2008). The *pADH:LUC* and *5xHRPE:LUC* Arabidopsis stable transgenic lines were produced as previously described (Triozzi et al. 2024). The *pADH:LUC* and *5xHRPE:LUC* reporter lines were screened and selected (3 independent lines) as described in Supplementary Fig. S3. Arabidopsis mutants *pdc1pdc2* (N660027 crossed with N862662) (Mithran et al. 2014) and *adh1* (N552699) obtained from the Nottingham Arabidopsis Stock Centre were also used in this study. Adult plants were grown in pots for 4 weeks at 23 °C with a 12:12 light:dark photoperiod at 120 µmol photons m^−2^s^−1^ intensity before being used for experiments. Seeds were stratified at 4 °C in the dark for 48 h and subsequently germinated at 22 °C day/18 °C night, with a 12:12 light:dark photoperiod. Protoplasts were isolated and transformed as previously described (Kunkowska et al. 2023). For in vitro experiments, seeds were surface-sterilized using a 70% (v/v) ethanol solution followed by incubation in a 1% (v/v) sodium hypochlorite and then washed several times with sterile water. Sterilized seeds were sown in either 6-well plates in liquid MS media (Murashige and Skoog half-strength medium, 0.5% w/v sucrose, 0.05% PPM (Plant Preservative Mixture), pH 5.7), or 96-well white plates as previously described (Triozzi et al. 2024). Seeds were stratified at 4 °C in the dark for 48 h and subsequently germinated at 22 °C day/18 °C night, with a 12:12 light:dark photoperiod, under continuous shaking conditions.

### Plant inoculation with *B. cinerea*

The *B. cinerea* strain (Ferrari et al. 2003) were cultured on MEP plates (maltose extract at 20 g/L, peptone 10 g/L, and phyto agar 15 g/L) for 15 to 20 d at 23 °C in the dark. The conidia were collected by washing the plate with potato dextrose broth (PDB, 12 g/L, Formedium^TM^), and the spore suspension was adjusted to the appropriate concentration (∼1 × 10^6^ conidia/mL). Inoculation with Botrytis was performed on 4-week-old plants by placing 5 μL droplets of the spore suspension on leaves. Inoculated plants were placed in a plastic tank and sealed with plastic transparent foil. The plants were then incubated in a growth chamber (Percival Scientific, Perry, IA, USA) at 23 °C with a 12:12 h light:dark photoperiod. Lesion size was measured using ImageJ software (http://rsb.info.nih.gov/ij/).

### Chemical treatments

For hormone treatments, a solution of 50 μM of MeJA (Merck KGaA, Germany) in 0.1% DMSO (Merck KGaA, Germany) was sprayed on the plants. Plants were then placed in transparent sealed tanks for 7 h before performing the experiments. For ethanol treatment, *B. cinerea* spores were resuspended in either PDB or PDB added with 100 mM Ethanol (Merck KGaA, Germany) before applying to the plants.

### Flooding, hypoxia and reoxygenation treatments

Plants (4-week-old) were submerged in a plastic tank in the dark while the relative controls were kept in the dark under normoxic conditions for the same amount of time. Hypoxic treatments were performed using an enclosed anaerobic workstation (Coy Laboratory Products) by flushing with an oxygen-modified atmosphere (1% [v/v] O_2_/N_2_) (Brunello et al. 2024). Plates (six-wells) with either seedlings or protoplasts were quickly opened inside the workstation at the beginning of the treatment to allow atmosphere exchange. The air controls were kept in the dark outside of the workstation, but they were also opened quickly to allow atmosphere exchange. For reoxygenation treatments 6-well plates containing 5-d old seedlings that were treated under hypoxia for 4 h were opened in a normoxic atmosphere to allow gas exchange. The recovery phase after reoxygenation was performed in the light at 120 µmol photons m^−2^s^−1^ intensity.

### Microarray analysis

Affymetrix GeneChip Arabidopsis ATH1 Genome Arrays were used to process RNA, as previously described (Loreti et al. 2005). Each of the samples was composed of pooled RNA extracted from 5 biological replicates. Hybridization, washing, staining, and scanning procedures were performed by Genopolis (University of Milano-Bicocca), as described in the Affymetrix technical manual. Microarray analysis was performed using an R/Bioconductor (Gentleman et al. 2004) and Robin (Lohse et al. 2010). MAS5.0 was used to preprocess the raw *.CEL data by performing background correction, data normalization, conversion of the original data, and quartile data normalization. The raw data are reported in Supplementary Table S1 as MAS5.0-normalized values. Normalized gene expression values were log_2_ transformed and subsequently analyzed (all subsequent analyses were performed using the log_2_-normalized gene expression counts as input). To visualize the expression levels of the core anaerobic genes after Botrytis infection, a bar plot was generated using the ggplot2 package in R. The plot (Fig. 1A) includes a dashed horizontal line at log_2_FC = 1, indicating the threshold for significantly upregulated genes under anaerobic conditions.

### Construct preparation

The *ADH* promoter (1,099 bp upstream the transcriptional starting site of *AT1G77120*), was amplified using Phusion High Fidelity DNA Polymerase (Thermo Fisher Scientific) from Arabidopsis genomic DNA using the primers listed in the Supplementary Table S2. The *ADH* promoter was cloned in the pENTR/D-TOPO entry vector following manufacturer's instructions (Life Technologies). 5xHRPE synthetic promoter was as previously described (Panicucci et al. 2020). To avoid autoactivation in Y2H assay, the coding sequences of *ERFVIIs* were cloned without their CMVII-5 domains and *ORA59* without its activation domain (ORA59Δ1–60) (Kim et al. 2018). The truncated *RAP2.12* (Bui et al. 2015), and the *HOL2* control construct were previously described (Carlessi et al. 2021). For split-LUC assays experiments, the full-length versions of ERFVII together with the truncated coding sequence of *ORA59* were used. The full-length version of ERFVIIs as well as of ORA59 were used in all the protoplast transformation experiments. The ERFVIIs were as follows: *RAP2.2* and *RAP2.3* (Bui et al. 2015), *RAP2.12* (Weits et al. 2014), *HRE1*, and *HRE2* (Licausi et al. 2010). These entry vectors were then recombined into destination vectors using the LR Clonase II Enzyme mix (Thermo Fisher Scientific) to create the corresponding expression vectors. A complete list of primers and vectors used for cloning is provided in Supplementary Table S2.

### LUC activity quantification

Firefly (*Photinus pyralis*) and *Renilla reniformis* LUC activities were quantified in Arabidopsis protoplasts using either the Dual-Luciferase Reporter Assay System (Promega) for transactivation assays or the Luciferase Reporter Assay System (Promega) for split-LUC complementation assays (Betti et al. 2021). In the transactivation assays, firefly LUC activity was normalized to *Renilla* LUC activity. In the split-LUC complementation assays, firefly LUC activity was normalized to the total protein concentration, which was quantified using the Bradford protein assay (Bio-Rad).

LUC activity of *35S:RAP2.12_1–28_-LUC*, *pADH:LUC*, and *5xHRPE:LUC* reporter lines was monitored in 7-d-old seedlings germinated into 96-well white plates. *35S:RAP2.12_1–28_-LUC* was used for the experiment reported in Fig. 6B. A solution of 5 mM of D-luciferin (Biosynth) was added to each well (30 µL per well), the day before the experiment. Plates were sealed with a transparent film as previously described (Triozzi et al. 2024). The TriStar5 LB 942 luminometer (Berthold Technologies, Germany) was used to measure the LUC activity.

In vivo LUC activity imaging was performed on 25-d old Arabidopsis plants using the NightSHADE system (Berthold Technologies, Germany) as previously described (Triozzi et al. 2024).

### Yeast two-hybrid and CPRG assays

The MaV203 *S. cerevisiae* strain was used (*MATα*; *leu2–3112*; *trp1–901*; *his3Δ200*; *ade2–101*; *cyh2^R^*; *can1^R^*; *gal4Δ*; *gal80Δ*; *GAL1::lacZ*; *HIS3_UASGAL1_::HIS3@LYS2*; *SPAL10*::*URA3*) (Thermo Fisher Scientific). Yeast co-transformation was performed following the LiAc-mediated transformation protocol from the Yeast Protocol Handbook (Clontech). Successfully co-transformed colonies were then inoculated in 5 mL of SD medium lacking leucine (Leu) and tryptophan (Trp) and grown overnight at 30 °C with shaking at 300 rpm. The next morning, 2 mL of yeast liquid culture was harvested and washed with distilled water. Subsequently, 4 µL of each suspension was added to SD media lacking Leu, Trp, and histidine (His) and containing 10 mM 3-amino-1,2,4-triazole (3-AT; Sigma–Aldrich). The plates were photographed after 3 d of incubation at 30 °C. In this assay, ORA59 was used as bait, and ERFVIIs were used as prey. All interactors were cloned without their activation domains to eliminate autoactivation (Kim et al. 2018). Positive and negative interaction controls were obtained from the ProQuest Two-Hybrid System (Thermo Fisher Scientific). The controls included bait pEXP32/Krev1 co-transformed with either pEXP22/RalGDS-wt (strong interaction; referred to as “pos. ctrl 1” in this study), pEXP22/RalGDS-m1 (weak interaction; “pos. ctrl 2”), or pEXP22/RalGDS-m2 (no interaction; “neg. ctrl”). A quantitative β-galactosidase assay was performed following the ProQuest^TM^ protocol (Thermo Fisher Scientific) using chlorophenol red β-D-galactopyranoside (CPRG; Sigma–Aldrich) as a substrate.

### RNA extraction and RT-qPCR analysis

Total RNA was isolated as previously described with minor modifications (omission of aurintricarboxylic acid to make the protocol compatible with the subsequent PCR procedures) (Perata et al. 1997). Electrophoresis using a 1% agarose gel was performed for all RNA samples to check for RNA integrity, followed by spectrophotometric quantification. cDNA synthesis and real-time quantitative PCR were performed as previously described (Loreti et al. 2020). Ubiquitin10 (*At4g05320*) was used as a housekeeping gene. The QuantPrime tool (Arvidsson et al. 2008) was used to design primers. The primers used for RT–qPCR are listed in Supplementary Table S2. Relative expression levels were calculated using geNorm (https://genorm.cmgg.be/).

### GUS staining

Histochemical GUS staining was carried out as previously described (Jefferson et al. 1987). Briefly, plant material was fixed immediately after sampling in ice-cold 90% acetone (Merck KGaA, Germany) for 1 h, rinsed several times in 100 mM phosphate buffer (pH 7.2), and then stained in a freshly prepared reaction solution (0.1% Triton X-100, 10 mM Na-EDTA, 0.5 mM potassium ferrocyanide, 0.5 mM potassium ferricyanide, and 1 mM X-Gluc [5-bromo-4-chloro-3-indolyl β-D-glucuronide, dissolved in DMSO] in 100 mM phosphate buffer, pH 7.2). The plants were stained overnight, and chlorophyll was removed from the green tissues by washing with 96% ethanol (Merck KGaA, Germany) before image acquisition.

### Protein extraction, SDS–PAGE, and immunoblotting analysis

For total protein extraction, pools of 25-d-old Arabidopsis infected leaves from plants were grinded in liquid nitrogen into a fine powder and mixed with 300 μL of protein extraction buffer per 100 mg fresh tissue (50 mM Tris-HCl pH 7, 1 mM EDTA pH 8, 100 mM NaCl, 2% SDS and 0,05% Tween-20 with Protease Inhibitor Cocktail, Sigma). Protein content was quantified with Pierce BCA Protein Assay Kit (Thermo-Fisher Scientific). Immunoblotting analysis and membrane visualization were performed as previously (Triozzi et al. 2024). Anti-HA peroxidase 3F10 antibody (Roche) at 1:1,000 dilution was used for the immunodetection of RAP2.3^3xHA^. Signal detection was performed with Clarity Max Western ECL Substrate (Bio-Rad), using a ChemiDocTM MP Imaging System (Bio-Rad). Band intensities were normalized based on the amido black staining (Merck KGaA, Germany). The original, uncropped immunoblot images are reported in Supplementary Fig. S4.

### Accession numbers

Microarray datasets were deposited in a public repository with open access (GSE270441; http://www.ncbi.nlm.nih.gov/projects/geo). Accession numbers of the major genes/proteins mentioned in the article are reported in Supplementary Table S2.

## Supplementary Material

kiae677_Supplementary_Data

## Data Availability

The data underlying this article are available in a public repository with open access (https://www.ncbi.nlm.nih.gov/geo) and can be accessed with accession number GSE270441.

## Supplementary data

The following materials are available in the online version of this article.

**Supplementary Figure S1.** Expression analysis of *PDF1.2* in Col-0 and *ora59* RNAi plants 48 h after inoculation with Botrytis.

**Supplementary Figure S2.** Representative images of Arabidopsis leaves with and without 100 mM exogenous ethanol.

**Supplementary Figure S3.** Luciferase activity in three independent lines of pADH:LUC and HRPE:LUC 7-days old seedling up to 8 h of gaseous hypoxia.

**Supplementary Figure S4.** Original images of the immunoblot and of the amido black staining.

**Supplementary Table S1.** Raw microarray data.

**Supplementary Table S2.** Accession numbers of the major genes/proteins mentioned in the article, primers list, cloning sequences and vectors.

## Dive Curated Terms

The following phenotypic, genotypic, and functional terms are of significance to the work described in this paper:

ORA59 Gramene: AT1G06160
ORA59 Araport: AT1G06160

CML38 Gramene: AT1G76650
CML38 Araport: AT1G76650

JAZ1 Gramene: AT1G19180
JAZ1 Araport: AT1G19180

SRO5 Gramene: AT5G62520
SRO5 Araport: AT5G62520

ADH1 Gramene: AT1G77120
ADH1 Araport: AT1G77120

LUC Gramene: luciferase
LUC Araport: luciferase

UBQ10 Gramene: AT4G05320
UBQ10 Araport: AT4G05320

RAP2.3 Gramene: At3g16770
RAP2.3 Araport: At3g16770

RAP2.12 Gramene: At1g53910
RAP2.12 Araport: At1g53910

PDC1 Gramene: At4g33070
PDC1 Araport: At4g33070
