# Supplementary material for: The transcription factor ORA59 represses hypoxia responses during Botrytis cinerea infection and reoxygenation
Source: Plant Physiol. 2024 Dec 20;197(1):kiae677. doi: 10.1093/plphys/kiae677 (PMC11707877; doi:10.1093/plphys/kiae677)
Supplement: kiae677_Supplementary_Data [file kiae677_supplementary_data.zip › pp.02909.2024-s08.pdf]

Plant Physiology®

The AP2/ERF domain transcription factor ORA59 represses hypoxia responses during Botrytis cinerea infection and reoxygenation

Luca Brunello, Alicja Kunkowska, Emma Olmi, Paolo Triozzi, Simone Castellana, Pierdomenico Perata, and Elena Loreti

Review Timeline:

Submission Date:	26-Jun-2024
Editorial Decision:	17-Aug-2024
Resubmission Received:	24-Nov-2024
Editorial Decision:	27-Nov-2024
Revision Received:	27-Nov-2024
Accepted:	28-Nov-2024

17-Aug-2024

Dr. Elena Loreti
Institute of Agricultural Biology and Biotechnology, CNR, National Research Council
Pisa
Italy

RE: ERF-VII transcription factors define the hypoxic and defence response trade-off during Botrytis cinerea infection

Dear Elena,

Thank you for giving us the opportunity to review your manuscript for publication in the Plant Physiology in the context of the Focus Issue on hypoxia. I have been able to secure an assessment of two expert reviewers. You will see that both have found the manuscript and the link between hypoxia siganlling and biotic stress of major interest, and I wholeheartedly agree. Yet, there has also been agreement with respect to a number of concerns that will require your attention. As these concerns are of some gravity and will entail substantial changes to the manuscript storyline as well as additional experimentation, you are likely to need additional time to address them. I must therefore reject the manuscript in its present form. Nonetheless, if you are able to address the points raised in review, I would welcome your resubmission of a manuscript that incorporates your responses.

I have found both reviews highly instructive and would like to refer you to the individual point-by-point assessments. Both reviews are generally supportive and appreciate the overall relevance and the timeliness of investigating the potential interaction between low oxygen and biotic stress signalling. Reviewer #1 makes several useful suggestions on how to tighten the storyline. I agree that is currently not particularly accessible and requires streamlining. In particular aspects that are not far developed in terms of data support (such as the proposed SA link) either deserve additional follwo up or may be taken out. Reviewer #2 asks a string of highly relevant questions that deserve to be clarified, not only in the form of a response, but also by re-working of the manuscript. It is highly likely that the readership will have similar thoughts. For a potential resubmission I would like to encourage you to focus on clarity and conclusiveness of the presented work and the interpretations.

Please note that any resubmission will be treated as a new entry to the system and is likely to go out for review.

If you do have any queries or concerns, you are welcome to contact me in the first instance.

Thank you again for allowing Plant Physiology to review your work. We look forward to hearing from you soon.

Sincerely,
Editor, Plant Physiology

-- Reviewer comments:
Reviewer #1 (Comments for the Author):

Brunello et al. aimed to understand the contribution of ERFVIIs transcription factors in plant defense strategy against Botrytis cinerea. The starting point is the susceptibility of erfvii mutants to the pathogen together with the previous observation from his group that a hypoxic niche is established near the lesion provoked by the fungus. I found the premises on which the manuscript is based on very interesting for the hypoxia and phytopathology field. However, in my understanding the authors tried to combine too many distinct regulatory pathways such as hypoxia, oxidative stress, jasmonic acid, ethylene and salycilic acid signaling in the same working model based, almost solely, on gene expression data. The story progresses to a somewhat confusing and complex working model in the end. Nonetheless, when considered in an isolated manner, several of the observations are very interesting.

The authors approached the problem by performing a microarray analysis of Col-0 and erfvii mutants infected with Botrytis. Particular focus was given to the Hypoxia Responsive Genes (HRGs) defined by Mustroph (2010). They observed two distinct clusters of HRGs: Group 1, transcripts that were up-regulated independent of the ERFVIIs and Group 2, transcripts that were not up-regulated in both lines after Botrytis treatment. One of the transcripts belonging from the Group 2 is the classical hypoxic marker Alcohol Dehydrogenase (ADH). From this observation the authors tried to depict the mechanism behind each of the two observations.

Major Revisions

1. There are several interesting findings in this article, however the storyline is too difficult to follow. This manuscript would benefit immensely if it was focused solely on, for example, how the HRGs from Group 1 are induced independent on the ERFVIIs. Alternatively, the ORA59-ERFVII interaction to repress HRGs from Group 2 during Botrytis infection is also very interesting. Deep investigation of any of those points would yield two important manuscripts for the hypoxia and phytopathology community. As the story is now, I would rather make it more concise focusing on the ERFVII-ORA59 node.

2. Lines 73-74: Although this is first mentioned at the introduction, this assumption guide the authors in their interpretation. However insufficient to trigger pPCO:GUS, Loreti et al. (2021) clearly observed an almost 30% decrease in oxygen concentration in the presence of the pathogen elicitor flg22. Veillet et al. (2017) also elegantly demonstrated that increase oxygen consumption in Arabidopsis occur independently of the establishment of Botrytis infection sites. An enrichment of HRGs can also be found in public transcriptome datasets of elicitor treated Arabidopsis plants, such as in the data published by Bjornson et al. (2021). Altogether, is the establishment of a local hypoxia detrimental to the cell and benefits the pathogen or just part of plant defense response?

3. Figure 1B, C. These figures do not seem relevant to the manuscript, since the authors decided to follow a completely distinct route. The transcripts that are not induced in the erfvii background are not mentioned in any point beyond in the manuscripts and also are not relevant in the way the story is being told.

4. Supplemental table 1 containing the microarray data is rather incomplete. It does not contain neither statistical analysis nor expression values. A complete table containing every single transcript together with the expression values for both genotypes should be provided at least to allow the reader to look at the data the manuscript is based on.

5. The methodology describing the time points and how the samples were collected for microarray are also strictly necessary for the interpretation of further results. It seems from some Figure legends from RT-qPCRs that this data comes from 48h post-infection, although it is not clear if this timing is maintained for the microarray dataset. Were whole leaves collect of only the regions close to the lesions?

6. L122-124 The evidence of a hypoxia response at transcript level is convincing based on all qRT-PCR data performed in combination of pPCO:GUS histochemical staining. However, evidence of ERFVII stabilization is not that convincing. The confocal microscope images shown by Loreti et al. previously lacks normalization which could create some bias in addition it comes from a low number of replicates. Imaging in combination with Immunoblotting would give the reader more convincing evidence for ERFVIIs stabilization in the lesion area, specially because the whole argument the manuscript is based on relies on ERFVIIs stabilization.

7. The authors proposed that H_2O_2 could mediate the up-regulation of HRGs in an ERFVIIs independent manner. Why does it look that, even though approx. 300mM H_2O_2 was used the transcriptional response was not that intense when compared to Botrytis treatment. Is there evidence that ROS formation is still pronounced at the time samples from the infected leaves were collected?

8. The authors propose that lack of ethanol production in the lines pdc1pdc2 and adh could be beneficial for plant defense strategy. What is the rationale that could explain the induction of PDC1 and PDC2 together with the repression of ADH, via ERFVII-ORA59 interaction, in Col-0 plants?

9. The use of genetic complementation in stably transformed Arabidopsis, for example in the erfvii mutants, would be much more reliable then protoplast transformation.

10. I would suggest to completely get rid of SA in the way the story is presented, unless SA is actually measured. It is hard to convince the reader based on a single marker gene.

12. The same rationale applies for ethanol production in the working model. Ethanol production was never measured.

13. Why the average lesion ratio in the Col-0 shown in Figure 5a is 6-fold increased when compared to Figure 1a?

Reviewer #2 (Comments for the Author):

EIN2 and ERFs appear to be important in resistance against Botrytis infection (necrotrophic fungus), particularly ORA59. ORA59 and RAP2.3 are also reported to be important in resistance against a different necrotrophic bacterium, Pectobacterium. A number of studies indicate that elevated RAPs are good for resisting infection; given Botrytis infection leads to local hypoxia due

to fungal respiration (reported in 2021), the authors propose this suggests a role for RAP members in response to infection. The paper seeks to understand the mechanism for this, including interactions with ORA59.

This paper seeks to build on emerging literature which indicates a role for ERFVIIs in response to infection, particularly via a link with ORA59 which is an ERFXI involved in resistance against Botrytis infection. The authors seek to understand the role of ERFVIIs in infection responses, particularly given the local hypoxia induced by Botrytis infection.
They start by using an erfvii mutant to demonstrate the role of ERFVII in resisting infection and ERFVII related gene upregulation in response to Botrytis infection. They then looked at the response of typical hypoxic response genes (HRGs) and surprisingly found that some HRGs were induced upon infection even in the absence of ERFVII (termed Group A) while many of them were not induced in response to infection (despite the fact that infection induces hypoxia); this latter subset were termed Group B.
To rationalise Botrytis-induced HRG expression in the absence of ERFVII, the authors proposed oxidative stress as a trigger. In Col-0, they used a pPCO1-GUS line exposed to H2O2 and found GUS expression, indicative of HRG promoter activation. They then examined Col-0 and erfvii plants treated with H2O2 or MV and analysed expression of selective oxidative stress genes or HRGs. These data indicated that a subset of HRGs can be upregulated by oxidative stress in a manner independent of ERFVIIs.

Q1 - This is an interesting finding, however the selection of a few genes to examine by qPCR following H2O2/MV treatment likely misses key information and currently the connection is a bit weak. Did the authors consider screening the expression of a wider selection of the HRGs following this oxidative treatment to confirm that those not upregulated by Botrytis were also not upregulated by H2O2? This may provide more information about the mechanism of ERFVII independent upregulation, which is currently a bit open-ended.

They next sought to rationalise why Botrytis did not cause upregulation of all HRGs, and looked at JA pathways induced by Botrytis infection. They report that JA treatment showed a 'much lower induction of HRGs when submerged' compared to untreated plants.

Q2 - The differences in HRG expression in MeJA-treated vs untreated are $P<0.05$, however one or two of the data points appear to overlap; I would caution against using the word 'much lower' as it is a little misleading. In addition, this set of HRGs were chosen to examine as they are in Group B, however might an informative control have included one or two HRGs from Group A as well?

Both JA and Botrytis were shown to induce ORA59, which has previously been shown to interact with RAP2.3. The authors used Y2H and split luciferase assays to demonstrate nicely that ORA59del1-60 seems to interact with other ERFVIIs (except HRE2). When an ora59 rnai line was infected, HRGs from Group B were induced upon infection rather than repressed/remaining the same, suggesting ORA59 represses HRG expression

Q3 -Figure 4F shows FTM1 expression being the same +/- Botrytis treatment in Col-0, however Figure 1D suggests downregulation of FTM1 in response to the same treatment. Can you rationalize this (maybe I misunderstand the way Fig 1D was generated)?

When ORA59 was overexpressed in protoplasts, it dampened the expression of HRGs which were otherwise induced by hypoxia. Overall, the connection between JA-mediated ORA59 activation and partial repression of HRG expression (at least of those tested) is strong.

Q4 - The rationale for picking these 5 genes to examine is not clear, and the connection would be even stronger with inclusion of additional Group B HRGs (and countered by Group A HRGs).

The authors then went on to consider whether activation of HRGs is detrimental to resistance to Botrytis infection using pdc1pdc2 and adh1 mutants. These plants were more resistant to infection, suggesting these genes are detrimental to resistance, possibly via ethanol effects. SA biosynthesis genes PR1 and SID2 were lower in these mutants. There is an interplay reported in the literature of the effects of JA and SA on ORA59, with SA resulting in ORA59 degradation. This was confirmed by the authors in a mutant lacking a key enzyme in SA biosynthesis. RAP2.12 on the other hand has been reported to promote SA biosynthesis; the authors observed repression of the SA biosynthesis gene in the erfvii mutant, which was in turn dampened by ERFVII. Overall, these observations allowed the authors to devise a model whereby ORA59 dampens the effects of ERFVIIs to promote Botrytis resistance.

This is an interesting study that reveals novel aspects of cross-talk between defence and hypoxia signalling through JA-induced ORA59 expression, which in turn represses HRG expression through interaction with ERFVIIs to reduce ethanol-mediated diminishing of infection tolerance. There is undoubtedly a lot that we still need to understand about cross-talk between different stress signalling pathways, and this report proposes a useful model that will need further validation in future studies.

The work would benefit from more secure relationships being established between the stimulus and the expression of the Group A and Group B HRGs, as described above. I was also intrigued that PDC1 and PDC2 are two of the Group A HRGs, increasing in expression upon infection; with ADH levels unchanged upon infection in Col-0 (according to Figure 1), could ethanol formation take place such that the detrimental impacts of ethanol on Botrytis infection tolerance may negate any advantage conferred by

JA-induced ORA59. The authors may wish to comment on this (maybe ADH levels are negligible?) or explore this further.

Minor comments
Line 96 - I would argue that it's better say 'ERFVII proteins are important for' rather than 'ERFVII proteins are required for' (their function could, in theory, be replaced by something else).
Improve annotation of supplementary material so there is consistent terminology between text and SI, e.g. in Supplementary Table 1, 'col up' tag label presumably corresponds to 'upregulated genes in Botyrtis-infected Col-0'.
Line 308 - can H2O2 concentration be given in uM to allow comparison to concentration of MV?
Figure 4 - please clarify why ORA59 has residues 1-60 deleted - is this the activation region mentioned in the Methods?

Reviewer #1 (Comments for the Author):

Brunello et al. aimed to understand the contribution of ERFVIIs transcription factors in plant defense strategy against Botrytis cinerea. The starting point is the susceptibility of erfvii mutants to the pathogen together with the previous observation from his group that a hypoxic niche is established near the lesion provoked by the fungus. I found the premises on which the manuscript is based on very interesting for the hypoxia and phytopathology field. However, in my understanding the authors tried to combine too many distinct regulatory pathways such as hypoxia, oxidative stress, jasmonic acid, ethylene and salycilic acid signaling in the same working model based, almost solely, on gene expression data. The story progresses to a somewhat confusing and complex working model in the end. Nonetheless, when considered in an isolated manner, several of the observations are very interesting.

REPLY: We agree with the reviewer. Consequently, we revised the manuscript by changing the focus of the manuscript on its main findings, namely the involvement of ORA59 and its interaction with the ERFVIIs and the consequent repression of their activity towards the activation of HRGs transcription.

The authors approached the problem by performing a microarray analysis of Col-0 and erfvii mutants infected with Botrytis. Focus was given to the Hypoxia Responsive Genes (HRGs) defined by Mustroph (2010). They observed two distinct clusters of HRGs: Group 1, transcripts that were up-regulated independent of the ERFVIIs and Group 2, transcripts that were not up-regulated in both lines after Botrytis treatment. One of the transcripts belonging from the Group 2 is the classical hypoxic marker Alcohol Dehydrogenase (ADH). From this observation the authors tried to depict the mechanism behind each of the two observations.

Major Revisions:

1. There are several interesting findings in this article, however the storyline is too difficult to follow. This manuscript would benefit immensely if it was focused solely on, for example, how the HRGs from Group 1 are induced independent on the ERFVIIs. Alternatively, the ORA59-ERFVII interaction to repress HRGs from Group 2 during Botrytis infection is also very interesting. Deep investigation of any of those points would yield two important manuscripts for the hypoxia and phytopathology community. As the story is now, I would rather make it more concise focusing on the ERFVII-ORA59 node

REPLY: We agree and focused the ms. on the ERFVII-ORA59 node as suggested by the reviewer.

2. Lines 73-74: Although this is first mentioned at the introduction, this assumption guide the authors in their interpretation. However insufficient to trigger pPCO:GUS, Loreti et al. (2021) clearly observed an almost 30% decrease in oxygen concentration in the presence of the pathogen elicitor flg22. Veillet et al. (2017) also elegantly demonstrated that increase oxygen consumption in Arabidopsis occur independently of the establishment of Botrytis infection sites. An enrichment of HRGs can also be found in public transcriptome datasets of elicitor treated Arabidopsis plants, such as in the data published by Bjornson et al. (2021). Altogether, is the establishment of a local hypoxia detrimental to the cell and benefits the pathogen or just part of plant defense response?

REPLY: We agree. We added a sentence about the work by Veillet et al. as well as reference to the fact that also flg22 indices a decrease in oxygen concentration (this both in introduction and discussion). Whether the establishment of local hypoxia is detrimental to the plant is an excellent question. Our results (including new ones described below) suggest that the repression of most of the ERFVII

dependent responses by ORA59 is positive for the plant, as it is hampering ethanol production through the combined action of PDC and ADH. Interestingly, we found the exogenous ethanol is detrimental to the plant cell, favoring Botrytis infection. We discuss this aspect in the new version of the manuscript.

3. Figure 1B, C. These figures do not seem relevant to the manuscript, since the authors decided to follow a completely distinct route. The transcripts that are not induced in the erfvii background are not mentioned in any point beyond in the manuscripts and also are not relevant in the way the story is being told.

REPLY: We agree. We removed this section.

4. Supplemental table 1 containing the microarray data is rather incomplete. It does not contain neither statistical analysis nor expression values. A complete table containing every single transcript together with the expression values for both genotypes should be provided at least to allow the reader to look at the data the manuscript is based on.

REPLY: The new Supplemental Table 1 includes all raw microarray data.

5. The methodology describing the time points and how the samples were collected for microarray are also strictly necessary for the interpretation of further results. It seems from some Figure legends from RT-qPCRs that this data comes from 48h post-infection, although it is not clear if this timing is maintained for the microarray dataset. Were whole leaves collect of only the regions close to the lesions?

REPLY: It is indeed 48h post-infection. We made this clear in the Figure legend as well as in the M&M section.

6. L122-124 The evidence of a hypoxia response at transcript level is convincing based on all qRT-PCR data performed in combination of pPCO:GUS histochemical staining. However, evidence of ERFVII stabilization is not that convincing. The confocal microscope images shown by Loreti et al. previously lacks normalization which could create some bias in addition it comes from a low number of replicates. Imaging in combination with Immunoblotting would give the reader more convincing evidence for ERFVIIs stabilization in the lesion area, specially because the whole argument the manuscript is based on relies on ERFVIIs stabilization.

REPLY: We agree. We performed an immunoblot demonstrating that RAP2.3 is stabilized by Botrytis infection (48h) in four independent biological replicates.

7. The authors proposed that H_2O_2 could mediate the up-regulation of HRGs in an ERFVIIs independent manner. Why does it look that, even though approx. 300mM H_2O_2 was used the transcriptional response was not that intense when compared to Botrytis treatment. Is there evidence that ROS formation is still pronounced at the time samples from the infected leaves were collected?

REPLY: This section was removed, in line with the new version of the ms. focusing on the ERFVII-ORA59 node.

9. The use of genetic complementation in stably transformed Arabidopsis, for example in the erfvii mutants, would be much more reliable then protoplast transformation.

REPLY: We complemented the erfvii mutant protoplasts with the five individual ERFVIIs to check for the impact of ORA59 on each of them. We certainly agree that stably transformed, complemented erfvii plants would be much better, but the contribution of ORA59 would then require also silencing/OE of ORA59 in each of the lines. This would be quite a lot of work. As far as OE of ORA59 is concerned, we struggled to obtain an OE line, which is however not ready yet. We believe that the multiple evidence we provided about ORA59 acting as a repressor also in plants and not only in protoplasts is robust. We showed that expression of HRGs is profoundly affected in the ore59 stably transformed plants (Fig.4A)

and also that inducing ORA59 by JA treatment leads to the expected repression of HRGs, including a stably transformed HRPE:LUC line (new experiment showed in Fig.2C).

10. I would suggest to completely get rid of SA in the way the story is presented, unless SA is actually measured. It is hard to convince the reader based on a single marker gene.

REPLY: Agreed!

12. The same rationale applies for ethanol production in the working model. Ethanol production was never measured.

REPLY: The working model was removed. The new version of the manuscript provides a straight-forward take-home message: ORA59 acts as a repressor of ERFVII.

13. Why the average lesion ratio in the Col-0 shown in Figure 5a is 6-fold increased when compared to Figure 1a?

REPLY: the intensity of Botrytis infection can vary among individual experiments. Fig.1A was removed, but some differences in the average lesion ratio are also present comparing Fig.5B and the new Fig. 5E. (with a more reasonable 3-fold difference between experiments.

Reviewer #2 (Comments for the Author):

EIN2 and ERFs appear to be important in resistance against Botrytis infection (necrotrophic fungus), particularly ORA59. ORA59 and RAP2.3 are also reported to be important in resistance against a different necrotrophic bacterium, Pectobacterium. A number of studies indicate that elevated RAPs are good for resisting infection; given Botrytis infection leads to local hypoxia due to fungal respiration (reported in 2021), the authors propose this suggests a role for RAP members in response to infection. The paper seeks to understand the mechanism for this, including interactions with ORA59. This paper seeks to build on emerging literature which indicates a role for ERFVIIs in response to infection, particularly via a link with ORA59 which is an ERFXI involved in resistance against Botrytis infection. The authors seek to understand the role of ERFVIIs in infection responses, particularly given the local hypoxia induced by Botrytis infection. They start by using an erfvii mutant to demonstrate the role of ERFVII in resisting infection and ERFVII related gene upregulation in response to Botrytis infection. They then looked at the response of typical hypoxic response genes (HRGs) and surprisingly found that some HRGs were induced upon infection even in the absence of ERFVII (termed Group A) while many of them were not induced in response to infection (despite the fact that infection induces hypoxia); this latter subset were termed Group B. To rationalise Botrytis-induced HRG expression in the absence of ERFVII, the authors proposed oxidative stress as a trigger. In Col-0, they used a pPCO1-GUS line exposed to H2O2 and found GUS expression, indicative of HRG promoter activation. They then examined Col-0 and erfvii plants treated with H2O2 or MV and analysed expression of selective oxidative stress genes or HRGs. These data indicated that a subset of HRGs can be upregulated by oxidative stress in a manner independent of ERFVIIs.

Q1 - This is an interesting finding, however the selection of a few genes to examine by qPCR following H2O2/MV treatment likely misses key information and currently the connection is a bit weak. Did the authors consider screening the expression of a wider selection of the HRGs following this oxidative treatment to confirm that those not upregulated by Botrytis were also not upregulated by H2O2? This may provide more information about the mechanism of ERFVII independent upregulation, which is currently a bit open-ended.

REPLY: this section of the ms. was removed. The focus of the new version of the ms. is the ORA59-ERFVII node, as requested by both reviewer 1 and the Managing Editor.

They next sought to rationalise why Botrytis did not cause upregulation of all HRGs, and looked at JA pathways induced by Botrytis infection. They report that JA treatment showed a 'much lower induction of HRGs when submerged' compared to untreated plants.

Q2 - The differences in HRG expression in MeJA-treated vs untreated are $P<0.05$, however one or two of the data points appear to overlap; I would caution against using the word 'much lower' as it is a little misleading. In addition, this set of HRGs were chosen to examine as they are in Group B, however might an informative control have included one or two HRGs from Group A as well?

REPLY: we edited the text to match the actual effects of MeJa. The new version of the ms. includes both genes of Group A and Group B.

Both JA and Botrytis were shown to induce ORA59, which has previously been shown to interact with RAP2.3. The authors used Y2H and split luciferase assays to demonstrate nicely that ORA59del1-60 seems to interact with other ERFVIIs (except HRE2). When an ora59 rnai line was infected, HRGs from Group B were induced upon infection rather than repressed/remaining the same, suggesting ORA59 represses HRG expression

Q3 -Figure 4F shows FTM1 expression being the same +/- Botrytis treatment in Col-0, however Figure 1D

suggests downregulation of FTM1 in response to the same treatment. Can you rationalize this (maybe I misunderstand the way Fig 1D was generated)?

REPLY: The HRGs "Group A", includes genes which are induced at least 2-fold by Botrytis. The criteria for inclusion in "Group B" is that they do not behave as hypoxia-responsive, despite hypoxia being present during Botrytis infection. The presence of hypoxia was not only demonstrated by Valeri et al. but is also supported by the new experiment in which we demonstrate that RAP2.3 is stabilized during Botrytis infection. To avoid any misunderstanding we removed the data on FTM1, which is replaced by a larger number of HRGs (10 HRGs studied by RT-qPCR vs. 5 in the previous ms.).

When ORA59 was overexpressed in protoplasts, it dampened the expression of HRGs which were otherwise induced by hypoxia. Overall, the connection between JA-mediated ORA59 activation and partial repression of HRG expression (at least of those tested) is strong.

Q4 - The rationale for picking these 5 genes to examine is not clear, and the connection would be even stronger with inclusion of additional Group B HRGs (and countered by Group A HRGs).

REPLY: We agree. We expanded our gene-expression analysis: 5 HRGs in the previous version of the ms. to 10 HRGs, 5 for each of the two groups (A and B).

The authors then went on to consider whether activation of HRGs is detrimental to resistance to Botrytis infection using pdc1pdc2 and adh1 mutants. These plants were more resistant to infection, suggesting these genes are detrimental to resistance, possibly via ethanol effects. SA biosynthesis genes PR1 and SID2 were lower in these mutants. There is an interplay reported in the literature of the effects of JA and SA on ORA59, with SA resulting in ORA59 degradation. This was confirmed by the authors in a mutant lacking a key enzyme in SA biosynthesis. RAP2.12 on the other hand has been reported to promote SA biosynthesis; the authors observed repression of the SA biosynthesis gene in the erfvii mutant, which was in turn dampened by ERFVII. Overall, these observations allowed the authors to devise a model whereby ORA59 dampens the effects of ERFVIIs to promote Botrytis resistance. This is an interesting study that reveals novel aspects of cross-talk between defence and hypoxia signalling through JA-induced ORA59 expression, which in turn represses HRG expression through interaction with ERFVIIs to reduce ethanol-mediated diminishing of infection tolerance. There is undoubtedly a lot that we still need to understand about cross-talk between different stress signalling pathways, and this report proposes a useful model that will need further validation in future studies.

The work would benefit from more secure relationships being established between the stimulus and the expression of the Group A and Group B HRGs, as described above. I was also intrigued that PDC1 and PDC2 are two of the Group A HRGs, increasing in expression upon infection; with ADH levels unchanged upon infection in Col-0 (according to Figure 1), could ethanol formation take place such that the detrimental impacts of ethanol on Botrytis infection tolerance may negate any advantage conferred by JA-induced ORA59. The authors may wish to comment on this (maybe ADH levels are negligible?) or explore this further.

REPLY: The question as to whether ethanol production can occur in the absence of ADH induction because ORA59 inhibits this classical response to hypoxia is a very relevant one. It would be impossible that ethanol is produced if ADH levels are not already sufficiently high under control conditions. We explored this possibility and found that ADH expression is already quite high in plants that were exposed to the high humidity utilized to allow Botrytis infection. This was demonstrated by using plants expressing GUS under the control of the promoter of ADH. These new results are shown in the new Fig. 5D. This experiment also confirms that there is no increase in the level of ADH during the 48h after infection, as predicted by the gene expression analysis. However, repression of induction of ADH is important for plant

tolerance to Botrytis. This is supported by two independent experimental pieces of evidence. First, mutants impaired in their ability to produce ethanol are more tolerant to Botrytis. Second, exogenous application of a droplet of very diluted ethanol was sufficient to reduce significantly Arabidopsis tolerance to Botrytis. Taken together, these results demonstrate that the basal level of ADH is sufficient for reducing the plant's tolerance, and that should ORA59 be unable to prevent ADH induction, the impact of negative ADH would be worst, given that hypoxia at the infection site would strongly induce ADH, boost ethanol production and leading to higher susceptibility to Botrytis.

Minor comments

Line 96 - I would argue that it's better say 'ERFVII proteins are important for' rather than 'ERFVII proteins are required for' (their function could, in theory, be replaced by something else).

REPLY: this part was removed from the text.

Improve annotation of supplementary material so there is consistent terminology between text and SI, e.g. in Supplementary Table 1, 'col up' tag label presumably corresponds to 'upregulated genes in Botyrtis-infected Col-0'.

REPLY: we apologise. We now provide the normalized raw data for the microarray, so that readers can analyze themselves the results.

Line 308 - can H2O2 concentration be given in uM to allow comparison to concentration of MV?

REPLY: this part was removed from the text.

Figure 4 - please clarify why ORA59 has residues 1-60 deleted - is this the activation region mentioned in the Methods?

REPLY: Yes, correct. We edited the M&M for clarity.

27-Nov-2024

Dr. Elena Loreti
Institute of Agricultural Biology and Biotechnology, CNR, National Research Council
Pisa
Italy

RE: The AP2/ERF domain transcription factor ORA59 represses hypoxia responses during Botrytis cinerea infection and reoxygenation

Dear Elena,

We are pleased to accept your manuscript for publication in the Plant Physiology. This acceptance is contingent on revision based on the comments of our reviewers. In particular, please consider the following:

I have been able to secure a review from one of the initial reviewers. They conclude that the manuscript is now improved and simplified thanks to the removal of the side stories about ethylene, oxidative stress and salycilic acid. In addition, the existence of an ORA59-ERFVII node during infection was reinforced by the addition of Figure 5 and 6.

There is one point of concenrn remaining which refers to the claim that fermentation is detrimental to the plant during infection. The exogenously applied ethanol in the new experiment (Fig. 5) raised some concerns. I believe this point can be addressed by a minor revision of the text by adjusting the wording and by modifying the discussion to include the previous study that the reviewer refers to.

Please highlight all changes and include a detailed annotation to changes to the text, with line numbers, and noting your responses to the comments.

To submit your revised manuscript, click:

Link Not Available

If you cannot return the revised manuscript within 8 weeks of receipt, please let us know. Otherwise, we will assume that you have elected not to revise the manuscript and are withdrawing it.

Thank you for allowing us to review your work. We look forward to hearing from you soon.

Sincerely,
Editor, Plant Physiology

-- Reviewer comments:
Reviewer #1 (Comments for the Author):

I have seen Brunello et al. manuscript in the first round. The authors decided to focus the manuscript on the ERFVII-ORA59 node and left aside for now data related to salicylic acid, ethylene and oxidative stress. This refocusing has improved both clarity and readability, resulting in a concise and objective narrative. Also, the authors implemented the suggestions made by both reviewers and the managing editor. I particularly appreciate the addition of the raw supplemental dataset, Figure 6 and changes in style such as in Figure 1. The manuscript now centers on the differential induction of hypoxia-responsive genes (HRGs) during Botrytis infection, categorized into two groups: GROUP A (induced) and GROUP B (not induced), despite the establishment of a hypoxic niche. The authors propose that the lack of GROUP B gene induction is linked to the interplay between jasmonic acid and hypoxia signaling, mediated by ORA59-ERFVII interactions. This hypothesis is supported by experimental evidence showing that ORA59 represses GROUP B HRGs, with these genes being upregulated during infection in the absence of ORA59. The ORA59-ERFVII node would be important for plant immunity, for example, by repressing fermentative metabolism. The authors indeed show that experimentally the absence of fermentation or exogenous applied ethanol alter tolerance to Botrytis.

Major comments

1) The authors tested whether fermentation has an impact on the success of Botrytis infection by applying exogenously ethanol (in response to Reviewer 2). It is stated as a response to Reviewer 2 that "a droplet of very diluted ethanol was sufficient to reduce significantly Arabidopsis tolerance to Botrytis. The authors state at L172-173 "the infection was performed in the presence of a physiologically-relevant ethanol concentration". I am not sure whether 100mM Ethanol (7%) is that low. Were lower doses tested and resulted in the same effect? The leaf treated with ethanol and subjected to infection (Figure 5E) looks different from the adh and other photos (there are patterns of yellowing distant from the infection site). Did treatment with ethanol in the absence of Botrytis resulted in any detrimental effect?
I understand the authors did this experiment to validate the correlation of fermentation with infection success. However, the same group nicely shown a few years before that the developmental penalty associated with the lack of ADH, PDC1 and PDC2 is greater during normal development then under hypoxia. This suggests that the pleiotropic effects of impaired fermentation during plant growth could lead to a broader metabolic rearrangement than what is captured within the limited scope of the experimental timeframe.
I would rather prefer discussion using previous data by the same group (Ventura et al., 2020) then making strong assumptions about the role of fermentation/ethanol in the infection at this point (unless additional controls are provided as supplemental material).

Minor comments
L114 - 117: Is the absence of HRA1 transcript accumulation enough to rule out its involvement? Would be safer to rephrase as "potentially rule out"
L128 - L129: The authors state "indicating that the MeJA repression is due to reduced ability of ERFVIIs to bind to the hypoxia-responsive promoter elements". Should the possibility of MeJA impacts ERVIIs stability be considered here?
L222-225: This sentence is too long and not clear.
Figure 6. Consider removing the lines from the C subpanel. Although it makes the graphs clearer it suggests erroneously that these dynamics in fact were observed.

Reviewer #1

Reviewer #1 (Comments for the Author):

Major comments

QUERY The authors tested whether fermentation has an impact on the success of Botrytis infection by applying exogenously ethanol (in response to Reviewer 2). It is stated as a response to Reviewer 2 that "a droplet of very diluted ethanol was sufficient to reduce significantly Arabidopsis tolerance to Botrytis. The authors state at L172-173 "the infection was performed in the presence of a physiologically-relevant ethanol concentration". I am not sure whether 100mM Ethanol (7%) is that low.

REPLY: We certainly agree that 7% would be a high concentration physiologically speaking. However, 100 mM equals 0.58% Ethanol (v/v) and is non-toxic to plants (Jackson et al. 1982). We edited the text removing "physiologically relevant".

QUERY Were lower doses tested and resulted in the same effect? The leaf treated with ethanol and subjected to infection (Figure 5E) looks different from the adh and other photos (there are patterns of yellowing distant from the infection site). Did treatment with ethanol in the absence of Botrytis result in any detrimental effect?

REPLY: Ethanol itself did not cause any effect on the leaves. We produced a new figure to represent adequately not only the effects after Botrytis infection but also all the controls (including ethanol 100mM alone). The text now includes this sentence: "*Leaves treated with ethanol-only did not show any toxicity symptoms, in line with the absence of ethanol toxicity per-se (Supplementary Fig. S2)*."

QUERY I understand the authors did this experiment to validate the correlation of fermentation with infection success. However, the same group nicely shown a few years before that the developmental penalty associated with the lack of ADH, PDC1 and PDC2 is greater during normal development then under hypoxia. This suggests that the pleiotropic effects of impaired fermentation during plant growth could lead to a broader metabolic rearrangement than what is captured within the limited scope of the experimental timeframe.
I would rather prefer discussion using previous data by the same group (Ventura et al., 2020) then making strong assumptions about the role of fermentation/ethanol in the infection at this point (unless additional controls are provided as supplemental material).

REPLY: There is probably a misunderstanding that originated from our wording in the Discussion. We added this sentence to the Discussion: "*Both ADH and PDC activities are required for optimal plant development, even under aerobic conditions (Ventura et al., 2020). Repression of ADH and PDC is therefore restricted to conditions inducing ORA59 expression, namely during pathogen infection and recovery from a previous hypoxic condition.*"

Minor comments

QUERY: L114 - 117: Is the absence of HRA1 transcript accumulation enough to rule out its involvement? Would be safer to rephrase as "potentially rule out".

REPLY: The text was rephrased as suggested.

QUERY: L128 - L129: The authors state "indicating that the MeJA repression is due to reduced ability of ERFVIIs to bind to the hypoxia-responsive promoter elements". Should the possibility of MeJA impacts ERVIIs stability be considered here?

REPLY: The data reported in Figure 6A indicated that RAP.2.3 is stable during reoxygenation, despite a burst in JA synthesis inducing ORA59. Additionally, Botrytis infection triggers a very clear RAP2.3 stabilization (Fig. 3A). Valeri et al. (2021) showed that the ERFVIIs are stable and localized in the nuclei during Botrytis infection. Together, this evidence, although indirectly, suggests that JA does not affect ERFVII stability.

QUERY: L222-225: This sentence is too long and not clear.

REPLY: The text was rephrased as suggested.

QUERY: Figure 6. Consider removing the lines from the C subpanel. Although it makes the graphs clearer it suggests erroneously that these dynamics in fact were observed.

REPLY: Curved lines represent polynomial fits passing through the mean of each group of observations, providing a visual representation of the trend within each genotype. We clarified this in the figure legend.

28-Nov-2024
Dr. Elena Loreti
Institute of Agricultural Biology and Biotechnology, CNR, National Research Council
Pisa
Italy

MSID: PP2024-RA-02909DR1
MS TITLE: The AP2/ERF domain transcription factor ORA59 represses hypoxia responses during Botrytis cinerea infection and reoxygenation

Dear Elena,

Thank you for your speedy resubmission after the request for minor corrections and your careful revision and clear response to the reviewer's comments.

Based on my own final inspection of the revisions, I am pleased to inform you that your revised manuscript "The AP2/ERF domain transcription factor ORA59 represses hypoxia responses during Botrytis cinerea infection and reoxygenation" has been accepted for publication in Plant Physiology. Your manuscript will be checked for consistency with journal formatting and image standards by one of our Science Editors, who will contact you shortly. They may have a number of queries and suggestions for you. Please keep in mind that they work to increase the online visibility of your article and its attraction for readers from the widest of backgrounds. You will have the opportunity to review any revisions, and I hope you will work constructively with them to get the most out of your research. You will also have the opportunity to review all changes when you receive the author proofs for your article.

Once validated for publication, your article will be sent to a Science Editor for a final review of your manuscript and source files. After your final files are uploaded, your article will appear in the next available issue and the online Preview version of your article will be posted after your signed Copyright and Checklist forms and final source files are received.

Please be sure to include the following in your manuscript file:
Harvard style citations
List of all author contributions and funding information at the beginning of manuscript after the titles and author list.
List all supplemental materials associated with the article, with titles, at the end of the ms, before the references and acknowledgments section.
Complete Figure Legends for all figures appearing in the article listed before the Literature Cited.

LICENSE INFORMATION Shortly after your paper is sent to production, you will receive an email from our publisher's author support team, **SciPris**, with information on article processing charges, as well as the types of Open Access license you would be able to purchase. The accepted version of your manuscript will not appear on our Advance Articles page until the license is selected and signed, so please look out for an email from SciPris. For more information see the Article Publication Fees section of our author guidelines: https://academic.oup.com/plcell/pages/General-Instructions

Finally, we encourage your submission of artwork for the journal cover. If there is an image or illustration related to your paper that you would like to have considered, please email it in .pdf or .tif format to clowe@aspb.org. An image title, brief, 2-3 sentence description of the image, and the name of the person credited for the image are also encouraged. If your image is selected to appear on the online cover of the journal, you will be contacted with the scheduled issue date and further information.

Thank you and congratulations on your Plant Physiology paper!

Sincerely,
Editor, Plant Physiology

=====
FIRST AUTHOR PROFILES
=====

We recognize the hard work that goes into being First Author of a Plant Physiology paper by publishing a "First Author Feature" for each First Author alongside your paper.

If the first author(s) would like to submit information for a feature on Facebook and Twitter, fill out the form by clicking on the link below:

Plant Physiology First Author Feature Form: https://forms.office.com/r/ZBCGZfhm8F
If you cannot access the form through the link above, please contact Rachel at rbelsky@aspb.org.

To upload an image of yourself to accompany your profile, please put your photo in the correct journal folder at the link below. You must label your image: Author First Name Last Name Article Title.

First Author Profile Image Dropbox: https://www.dropbox.com/scl/fo/xz029p7qjr2jml2rp2kmq/h?dl=0&rlkey=6fkxcaqmxx35ap152b2osy1fz

=====
IMPORTANT REMINDER: PEER REVIEW REPORTS
=====

If you opted to publish a peer review report along with your article during the original submission process, it will be prepared by the editorial staff and publicly posted with your manuscript, inside the zip file that contains any other supplemental material. As a reminder, the peer review report is a public record of all comments from editors and reviewers, as well as your prior responses, as you received them in the decision letters for each draft of your manuscript. If you agreed to publish this report and have changed your mind, or are not sure if you selected this option, please contact the editorial office as soon as possible before signing the license agreement from our publisher.
