# Supplementary material for: The transcription factor ORA59 represses hypoxia responses during Botrytis cinerea infection and reoxygenation
Source: Plant Physiol. 2024 Dec 20;197(1):kiae677. doi: 10.1093/plphys/kiae677 (PMC11707877; doi:10.1093/plphys/kiae677)
Supplement: kiae677_Supplementary_Data [file kiae677_supplementary_data.zip › PP2024RA02909DR1_Supplemental_Figure.pdf]

SUPPLEMENTARY FIGURES

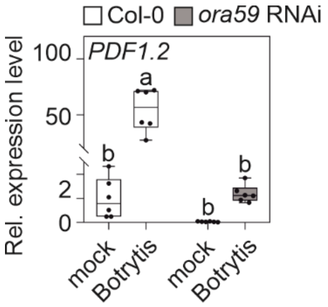

Supplementary Figure S1. Expression analysis of *PDF1.2* in Col-0 and *ora59* RNAi plants 48h after inoculation with Botrytis. Data (n=6) are relative to the Col-0 mock set to 1. Different letters indicate significant difference (p<0.05) after two-ways ANOVA followed by Tukey's post-hoc test.

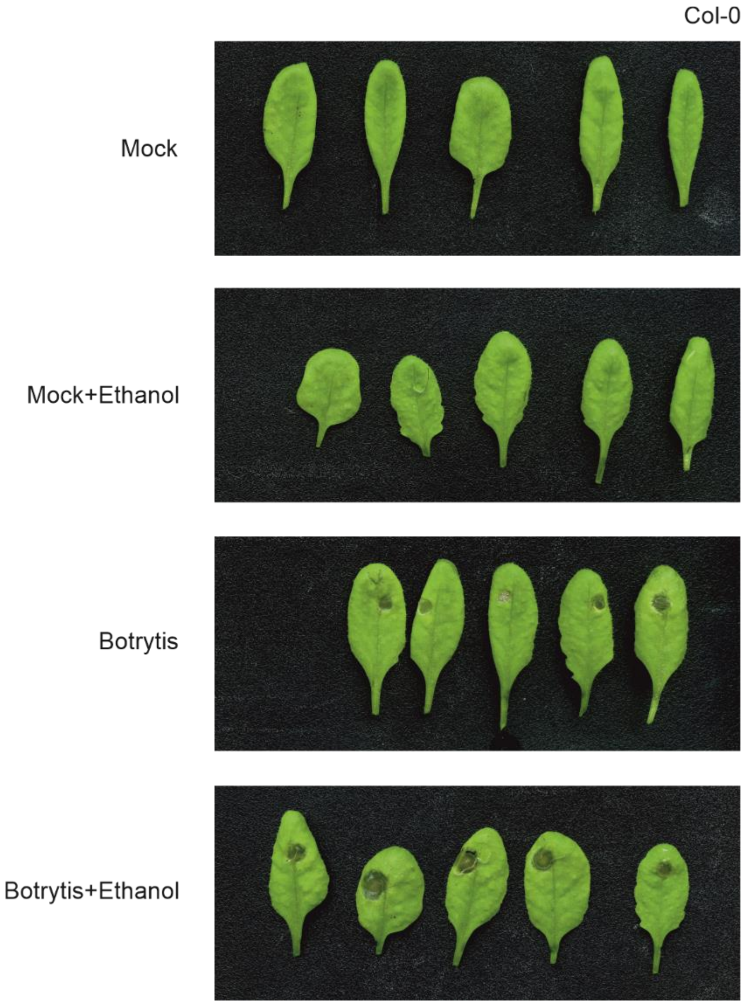

Supplementary Figure S2. Representative images of Arabidopsis leaves with and without 100mM exogenous ethanol. Infection with *Botrytis cinerea* was performed as described in the Materials & Methods section.

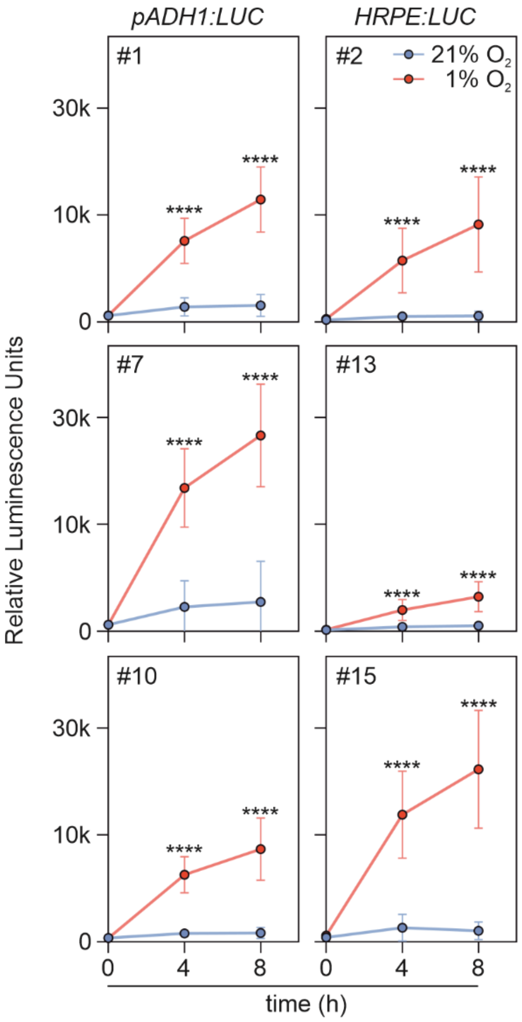

Supplementary Figure S3. Luciferase activity in three independent lines of *pADH:LUC* and *HRPE:LUC* 7-days old seedling up to 8h of gaseous hypoxia. Data (n=12) are presented in a graph and statistically significant differences are assessed using TWO-ways ANOVA followed by Sidak's multiple comparisons test (* = $p < 0.05$; ** = $p < 0.01$; *** = $p < 0.001$; **** = $p < 0.0001$). Lines #7 and #15 were selected for the experiments reported in Fig. 2C.

Immunoblot

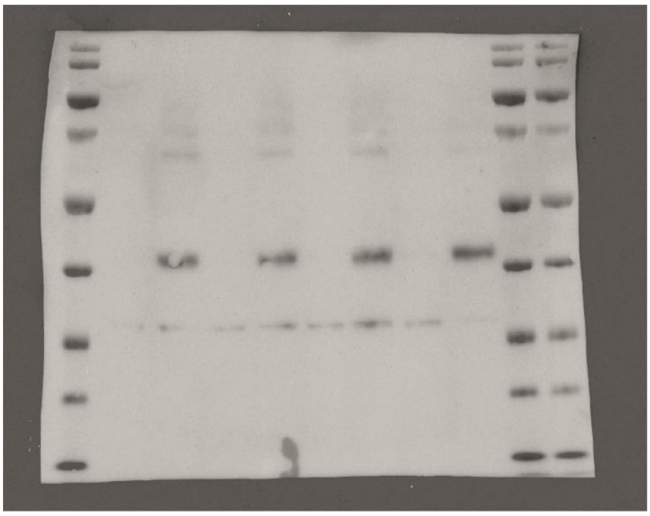

Amido Black

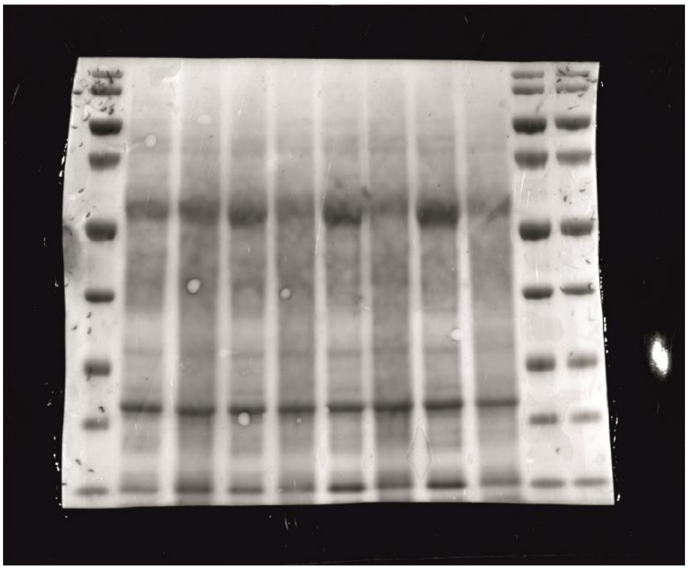

Supplementary Figure S4. Original images of the immunoblot and of the amido black staining.
